# Spatial Distribution and Hierarchical Behaviour of Cattle Using a Virtual Fence System

**DOI:** 10.3390/ani14142121

**Published:** 2024-07-20

**Authors:** Silje Marquardsen Lund, Johanne Holm Jacobsen, Maria Gytkjær Nielsen, Marie Ribergaard Friis, Natalie Hvid Nielsen, Nina Østerhaab Mortensen, Regitze Cushion Skibsted, Magnus Fjord Aaser, Søren Krabbe Staahltoft, Dan Bruhn, Christian Sonne, Aage Kristian Olsen Alstrup, John Frikke, Cino Pertoldi

**Affiliations:** 1 Department of Chemistry and Bioscience, Aalborg University, Fredrik Bajers Vej 7H, 9220 Aalborg, Denmark; jjac21@student.aau.dk (J.H.J.); maniek21@student.aau.dk (M.G.N.); mfriis21@student.aau.dk (M.R.F.); natani21@student.aau.dk (N.H.N.); nomo21@student.aau.dk (N.Ø.M.); rskibs21@student.aau.dk (R.C.S.); maaser20@student.aau.dk (M.F.A.); sstaah20@student.aau.dk (S.K.S.); db@bio.aau.dk (D.B.); cp@bio.aau.dk (C.P.); 2 Department of Ecoscience, Aarhus University, Frederiksborgvej 399, 4000 Roskilde, Denmark; cs@ecos.au.dk; 3 Department of Nuclear Medicine & PET, Aarhus University Hospital, Palle Juul-Jensens Boulevard 99, 8200 Aarhus, Denmark; aagealst@rm.dk; 4 Department of Clinical Research, Aarhus University, Palle Juul-Jensens Boulevard 99, 8200 Aarhus, Denmark; 5 Wadden Sea National Park, Havnebyvej 30, 6792 Rømø, Denmark; jofri@danmarksnationalparker.dk; 6 Aalborg Zoo, Mølleparkvej 63, 9000 Aalborg, Denmark

**Keywords:** cattle, virtual fencing, Nofence©, social hierarchy

## Abstract

**Simple Summary:**

In recent years, the interest in virtual fencing systems for flexible animal enclosure management has increased. However, implementing such systems requires significant investment due to the need for individual collars, hindering large-scale adoption. This study examines the spatial distribution of a cow herd using GPS locations from the Nofence© system, aiming to minimize the number of collars required by identifying patterns in positions and ranks to derive a hierarchy. Contrary to expectations, no distinct pattern emerged, suggesting future studies should focus on individual interactions rather than viewing the herd as a single unit.

**Abstract:**

Interest in virtual fencing has increased due to its flexibility for agriculture and rewilding. However, systems like Nofence© require large financial investments, and the need for individual collars complicates large-scale use. If cattle herds maintain cohesive groups around leading individuals, fewer collars could be used, thereby enhancing cost efficiency. This study investigates the pattern in spatial distribution in a herd of 17 Angus cows on Fanø in Denmark with GPS locations, using a Nofence© system. The aim of this paper is to determine how individuals position themselves in a herd, spatially, and identify a pattern in ranks. The method used in this study examines the distances between an individual to the rest of the herdmates using nearest neighbour and descriptive statistics. Contrary to expectations, this study did not reveal a distinct pattern in herd distribution. While some tendencies in spatial distribution patterns were observed, only a low concordance could be found (W=0.15,p<0.001), indicating great variability in the cattle’s ranks. A cumulative curve of the ranks estimated over the entire periods, however, allowed a rough estimation of the hierarchy and allowed identification of the highest-ranked cows, making the use of a cumulative curve a possible solution to finding the high-ranked cows. This research underscores the complexity of cattle social structures and highlights the need for extended observation periods and alternative methodologies to enhance the cost-effectiveness and scalability of virtual fencing in agricultural and rewilding contexts.

## 1. Introduction

Within the last century, humans have drastically accelerated the loss and alteration of biodiversity across the globe due to overexploitation of biological resources and the destruction of natural habitats [1,2]. Rewilding projects have been initiated throughout Europe, which is in line with the EU Biodiversity Strategy for 2030, aiming to protect and restore nature [2,3]. The aim of rewilding is to manage ecological succession with the objective of restoring ecosystems and ecological dynamics, thus enhancing biodiversity [1,4]. This can be achieved by reintroducing key grazers, such as cattle, sheep, or horses, to areas with high habitat homogeneity [1,4]. In areas of rewilding, barriers are needed to keep the large grazers in an enclosure to prevent conflicts between humans and animals [5,6]. These barriers are often physical, such as electric fences or sisal ropes, which can be obstacles for the local wildlife as they restrict their migration and are not fit for quick alterations [5,7,8,9]. As an alternative, virtual fences provide a solution that does not affect wildlife. By establishing borders that can be easily moved, virtual fences also reduce the risk of an area being overgrazed [10,11]. There are several types of virtual fence [8,10]. One of them is Nofence©. The Nofence© system works by equipping each animal with a collar. The collar is connected to a GPS-based system, and can issue auditory warning cues or electric impulses to keep the animals within a designated area. The system is primarily used on cattle and sheep, which have shown the ability to respond to the audio warnings issued by the collars, and the system has thereby proven to be successful at keeping the animals enclosed [12,13,14,15,16,17]. Therefore, Nofence© can also be beneficial for agricultural use, as it allows farmers to contain and move animals within certain areas more easily [10,13]. However, implementing the Nofence© system, might be considered costly and requires large financial investments from farmers to enclose their livestock [17]. The initial investment could have the potential of keeping buyers away, even though it could be cheaper in the long run, as most farmers already have their cattle enclosed within traditional physical fencing [10]. Also, a collar for every cow could make it financially difficult for farmers to use the Nofence© technology on a large scale [10,18]. To lower costs, it may be possible to reduce the number of collars by only placing them on leading herd members, assuming the herd maintains a cohesive social group around these individuals.

Previous studies show how cattle exhibit a tendency to form and maintain cohesive social groups as part of their normal behavior [19,20,21]. All cows within the herd may not require collars if they are organized in social groups around one or several leading individuals [19,22]. During grazing, cattle tend to fall into different movement styles: leading, following, and independent. More dominant cows have been found to have a higher tendency to lead the herd, while medium- and lower-ranking cows show more following and independence behavior, respectively [23]. It could then be sufficient to place a collar on the dominant individuals as the rest of the herd might follow these individuals, while also collaring individuals showing high independence. According to Arave and Albright [19], the dominant individuals occupy the middle during free movement, whereas Šárová et al. [22] conclude that the dominant individuals occupy the front during movement. The results from Šárová et al. [22] furthermore show that an increase in dominance leads to a greater influence on herd movement. Despite their different conclusions, all three studies suggest a pattern in the spatial distribution in relation to leadership and dominance. Studies such as those by Kondo et al. [24,25] and Šárová et al. [22] investigate behavior to describe such hierarchies and individual positional preferences based on the location of the herd and observation of individuals. However, this method is time-consuming and difficult to implement on a large scale [18]. Therefore, another method is needed to determine how the herd positions itself, and whether the herd follows one or more individuals. Current research has predominantly focused on the technical aspects and immediate behavioral responses of livestock to virtual fences [14,15,16,26,27,28]. However, there is a notable gap in understanding the long-term spatial dynamics and social interactions within cattle herds under such systems. While dominance and leadership are more complex than can be analyzed simply with distances, this paper aims to gauge an individual’s influence on the herd’s spatial behavior as a means of estimating leadership.

The aim of this paper is to determine how individuals within a herd position themselves in relation to each other, solely by looking at distances between individuals within a Nofence© system. This may allow farmers to reduce costs by only collaring leaders and independent cows. It is hypothesized to find an established pattern within the herd, with individuals maintaining a consistent position in relation to herd mates over the course of a given time period. The pattern is expected to show a few individuals with high or low ranks while the remaining cattle vary within the middle rank values [19,22]. The ranks are based on the distances between individuals. Identifying such patterns could provide a deeper understanding of spatial distribution, and thereby herd behavior, when using a Nofence© system. To investigate if any pattern appears, this paper examines the distances between an individual to the rest of the herdmates using nearest neighbour and descriptive statistics.

## 2. Materials and Methods

### 2.1. Location and Animals

**Figure 1:**
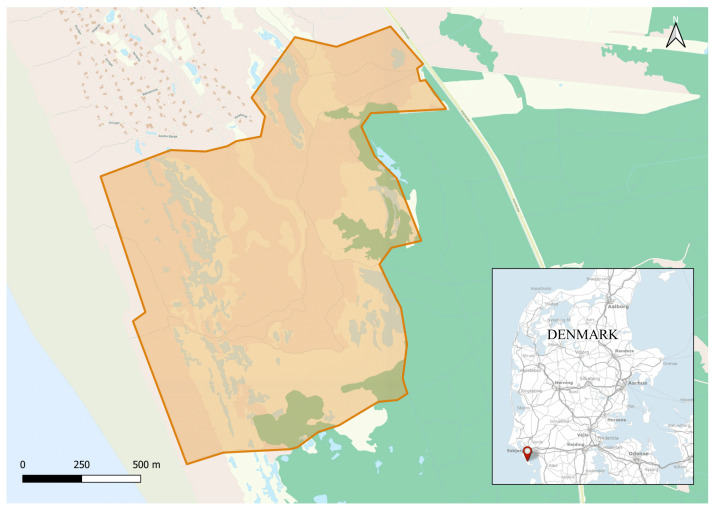
Map illustrating the study area Gåsehullerne on Fanø where the cattle were located during the period of observation. The orange lines mark the virtual fence borders and enclose an area of 160 ha [29]. Contains data from The Danish Agency for Data Supply and Infrastructure, https://dataforsyningen.dk/map/2683 (accessed on 5 December 2023). In the map, coastal moorland is marked with brown and dune landscapes with yellow.

The study was conducted on a herd of domestic cattle in an area called Gåsehullerne on the island of Fanø in Denmark (Figure 1). The cattle were located on the western part of the island in an area of coastal moorland and dune landscapes with grass and heath vegetation. The cattle had access to both lakes and dry areas covering a total of 160 hectares.

The herd was introduced to the area in May 2023. Data collection for this study took place between 15 August and 15 October 2023, during which the borders remained unchanged, and the cattle were well habituated to the area and the borders prior to data collection. The herd was kept for animal husbandry use and was not selected specifically for this study. This herd was additionally a part of a bigger study of testing virtual fencing in Denmark, which was approved by the Animal Experiments Inspectorate of Denmark. No changes were made to the herd or their situation to fit our study. The herd consisted of 17 Angus cattle (*Bos taurus*) (16 heifers aged 10–17 months and a single cow aged 11 years and 3 months). Throughout this paper, these animals will be referred to as cows.

### 2.2. The Nofence© System

All animals wore a Nofence© collar (Appendix A), each with a unique serial number, used to identify each individual (Appendix B). Of 17 animals, 16 wore the same collars throughout the entire experiment. One collar lost connection and was replaced after three days, and therefore, one animal had two serial numbers (Appendix B). The Nofence© collars were equipped with a battery, two solar panels, and an integrated GPS receiver. The GPS receiver utilizes the GNSS positioning system (GPS and GLONASS) meaning the signals have an accuracy of 3.5–10 m (GPS accuracy = 3.5–7.8 m, GLONASS accuracy = 5–10 m).

The collars issued three kinds of messages: a poll message, a client warning, and a client zap. All the messages consisted of a timestamp, a serial number, and longitude and latitude. The poll messages were issued every 15 min. In contrast to other messages, such as warnings, poll messages rely on mobile coverage, meaning these messages will be lost in situations with poor connectivity. The client warnings were issued with an auditory warning whenever an individual drew near the virtual border. The warning was a rising tone at 82 decibels lasting between 5 and 20 s, depending on whether the animal turned around or continued being at risk of crossing the virtual border. The client zap messages were issued if an individual continued its course toward the border despite the audio warning. The signal was paired with an electric shock of 0.2 J for one second. If the individual still did not change its direction another auditory warning would be issued followed by a shock warning. An individual can be given up to three electric shocks, thus sending three client zap signals before the warning system is disabled. The warnings are disabled until the animal reenters the enclosure where it reactivates.

#### Analysis of GPS Accuracy

To make sure the accuracy of the GPS locations was suitable for an analysis of herd behavior, a preliminary analysis was undertaken. This involved randomization of the distances to simulate situations where the data were biased by inaccuracy, and to test to what extent the accuracy affected the results. A total of 10 simulations of the randomized data were made. For each simulation, cumulative curves of the randomized data were made for comparison with cumulative curves of the non-randomized data. Additionally, pairwise comparisons were made of all the randomized medians of the hierarchical positions between all the cows with the Mann–Whitney U test. This was also performed between the randomized data and the non-randomized data to examine if significant differences occurred between the data sets. Furthermore, an analysis was performed to evaluate how significantly different the ranking of each cow was from others in both the original data and the randomized data set. This analysis also utilized the Mann–Whitney U test to identify any significant differences.

### 2.3. Data Collection

The timing of the poll messages was not aligned, meaning the poll signals came at a different time point for each individual in the herd. Prior to the data collection, alignment of the messages was pursued; however, the efforts had no effect. Therefore, it was necessary to arrange the data in 15 min intervals. Each day started with the interval at 00:00–00:15 UTC. The GPS signals from the client warnings and the client zaps were included in the data set. If several signals were issued from one cow within the 15 min interval, medians of longitude and latitude were taken. Some days were excluded from the data collection. A total of 16 days (22 August to 29 August, 1 September to 3 September, and 24 September to 28 September) were removed from the data set since less than 17 cows were registered. This could be due to the collars losing power or connection. In total 72,769 observations were collected across all 17 individuals (Appendix C).

### 2.4. Statistical Analysis

All data were sorted, and the statistical analysis was performed in R version 4.3.2 [30]. The following packages were used for data sorting and statistical purposes: Tidyverse version 2.0.0, Lubridate version 1.9.3, Raster version 3.6-26, Moments version 0.14.1, and DescTools version 0.99.54 [31,32,33,34,35]. For visualization, the package ggplot2 version 3.4.4 was applied [36]. Non-parametric methods were used for the statistical analyses, as the data did not follow a normal distribution. Furthermore, parametric methods require an assumption of the data being independent, which is rarely the case when analyzing spatial data.

The distance between the cattle was investigated by running a nearest neighbour analysis. For each 15 min interval, a distance between each individual was calculated, resulting in 16 distances per cow. This was performed using GPS positions derived from the different messages. Based on the 16 distances, a median was derived and used to determine the cow’s median distance to the rest of the herd. Each median value was ranked from the shortest (Rank 1) to the longest distance (Rank 17). For each day in the study period, a median was again calculated by grouping all ranks for every 15 min interval. This resulted in one single value representing how a cow ranked each day, where the lowest value indicates a high rank, and the highest value indicates a low rank. The use of a median-based method was chosen based on the amount of data being inconsistent between each cow throughout the study period, as the collars regularly lost signal for short periods of time.

To see how ranks were distributed between the cattle during the time period, a boxplot and a density plot were constructed. Both plots contain the rank for each individual each day. To analyse the density plot, descriptive statistics were used. Skewness and kurtosis were used to describe the shape of the curves. Skewness indicates if the data are symmetric. The thresholds used in this study were ±0.5 for skewed data and ±1 for extreme skewed. In this study, a positive skewness is referred to as right-skewed and a negative skewness as left-skewed. Kurtosis indicated how peaked the distribution is. A normal distribution has a kurtosis of 3, above 3 the distribution is peaked, and below 3 the distribution is flat. The peaks of the distribution were found to measure the most occurring rank for each cow. Peak x-values show the most occurring rank, while peak y-values indicate the density of the data that corresponds to each rank. The Spearman rank correlation was used to examine if there was a pattern in the skewness and peak x-values. In addition, a time series graph was used to visualize if any pattern in ranks occurred during the period, thus determining whether the cows maintained the same rank on a daily basis. A cumulative graph of ranks for each cow was also created and assessed as another way to visualize a possible pattern. Kendall’s coefficient of concordance (*W*) was calculated to test if such a pattern was significant [37].

## 3. Results

### 3.1. Analysis of GPS Locations

The cumulative curves of the randomized data showed that the cumulative ranks had a total range of 393.38–416.50 (Appendix D).

Pairwise tests of all the randomized medians of the hierarchical positions between all the cows with a pairwise Mann–Whitney U test were performed. None of these proved significant (Appendix E). The Mann–Whitney U tests between the original data set and the randomized data sets all proved significant (Appendix F).

Examining how significantly different the ranking of each cow was compared to other individuals for the original data and the randomized data resulted in mainly significant differences for the original data. The randomized data resulted in mainly insignificant *p*-values (Appendix G).

### 3.2. Analysis of Spatial Distribution

**Figure 2:**
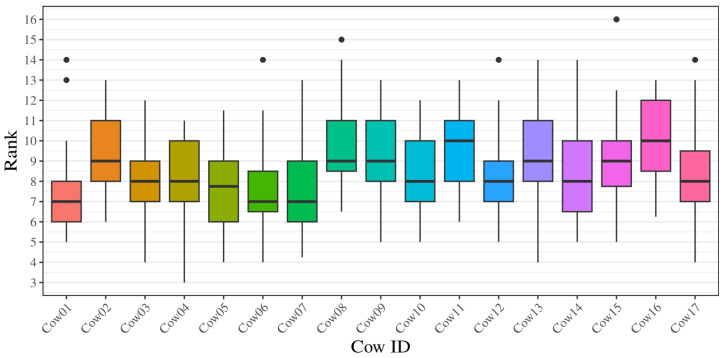
Boxplot of individual ranks based on median values for each of the 17 cows during the 48-day study period. Each data point represents the calculated rank a cow was found to have on a specific day, in relation to distances between the individual and the herd. The different boxplots represent a cow. The plot shows min, Q1, median, Q3, and max. Outliers are shown for Cow01, Cow06, Cow08, Cow12, Cow15, and Cow17.

The ranking distribution for each cow throughout the entire period lies within a minimum 3 and maximum 16 (Figure 2). The medians range between 7 and 10 (Figure 2).

**Figure 3:**
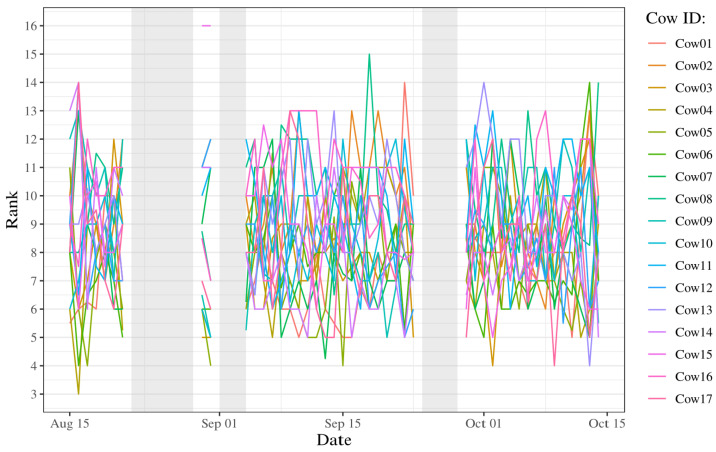
Times series of ranks for the 17 cows based on medians within the herd throughout the 45-day study period. Each color represents an individual cow in the herd. The period of study started on 15 August and ended on the 15 October. The grey marked areas show the excluded dates, resulting from signal issues.

As none of the cows maintained the same rank during the study period, the time series shows crossings and overlaps of the ranks between different individuals (Figure 3, Appendix H). Kendall’s coefficient of concordance shows a concordance in rank between the days (p<0.001). However, the concordance shows only a weak agreement (W=0.15), meaning that the ranks varied between days.

**Figure 4:**
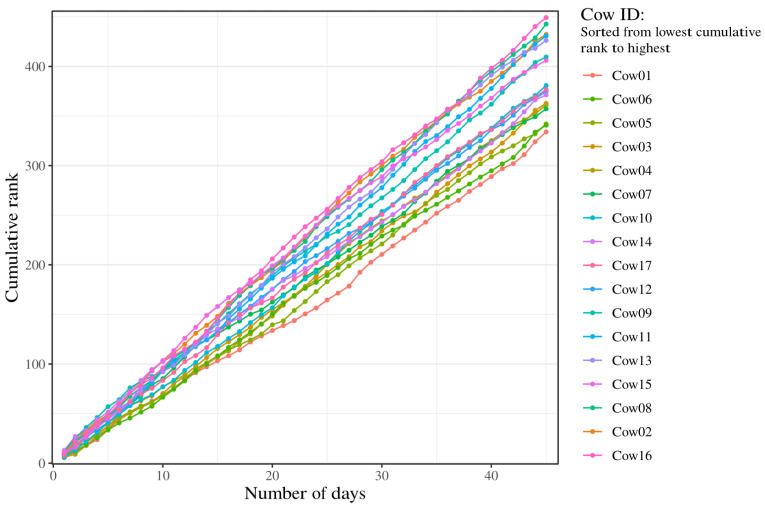
Cumulative rank for each cow in the 45-day study period. Each color represents an individual in the herd. The Cow ID is sorted in ascending order from lowest to highest cumulative rank. A low cumulative rank indicates an overall high rank. Cow01, Cow05, and Cow06 showed the lowest cumulative ranks and could, therefore, be considered the best candidates.

When assessing the cumulative rank of each cow, it shows an almost parallel course where the lines cross and overlap (Figure 4). A separation begins around day number 20, which increases slightly throughout the rest of the study period. At the conclusion of the study, the individual with the highest cumulative daily median rank attained a score of 449, indicating the least influence on herd cohesion. Conversely, the individual with the lowest cumulative daily median rank achieved a score of 334.

**Figure 5:**
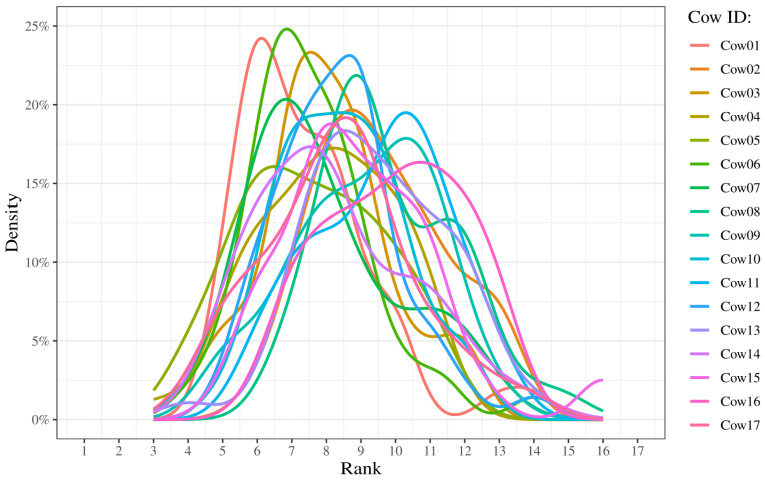
Density plot of ranks based on median values for each of the 17 cows during the 45-day study period. Density is represented with percentages illustrating the amount of times an individual is observed in a specific rank. Each color represents a single individual in the herd.

The distribution of ranks for each cow is compared to visualize how frequently the cows have a certain rank (Figure 5, Appendix I). All cows exhibit a broad span of ranks lying between 3 and 16, with peaks between 6 and 11 on all graphs (Table 1).

The graphs have peaks between rank 6 and 11, the skewness values range between −0.33 and 1.33, and the kurtosis values range between 1.91 and 5.09 (Table 1). Four cows exhibit a kurtosis value above 3, while 13 cows exhibit a kurtosis value below 3. The Spearman rank correlation shows a moderate negative correlation (ρ=−0.53) between the peak x-value and the skewness (Table 1).

## 4. Discussion

### 4.1. Accuracy of GPS Locations

To determine if the collected GPS locations were suitable for analysing the spatial distribution, the accuracy was analyzed by comparing the randomized data with the original data. The cumulative curves of the randomized data showed that the cumulative ranks had a range of 393.38–416.50, which is considerably shorter compared to the range found for the cumulative curves of the original data set (range of 334.00–449.25). The considerably lower range found for the randomized data compared to the range found for the original data set confirms that the cumulative curves plotted in Figure 4 cannot be due to random data. Examining the result of the different Mann–Whitney U tests, it is clear that the original data are not strongly influenced by inaccuracy. All the *p*-values between the randomized data and the original data were significant, which indicates a difference between the original data and the simulated situation of high inaccuracy. The matrices of the Mann–Whitney U tests between the individual cows for each data set further support that the data are not strongly influenced by inaccuracy. In all the data sets, *p*-values above and below 0.05 are found. However, a clear difference is seen, where the original data show many significant differences, while most of the random samples were non-significant. Comparing all of these results of the original data and the simulations of inaccuracy proves a clear difference, which indicates that the collected data used for the analysis of spatial distribution are not due to chance. The accuracy of the GPS positions can, therefore, be considered high enough to allow us to consider the distance between the GPS positions to be reliable.

### 4.2. Pattern in Spatial Distribution

To determine whether any pattern was evident in the spatial distribution, correlations of ranks throughout the study period were calculated. The ranks in this study were based on distances derived from a nearest neighbour analysis. The expectation was to see a pattern that shows few individuals with high or low rank, with the remaining cattle varying within the middle rank values during the period. The expectation was based on the placement of the leading individuals as a herd moves freely [19,22]. However, this study does not show a pattern in the spatial distribution and, therefore, no obvious leader was found within the herd using this method. The time series shows great variation in the computed ranks throughout the study period. This means none of the cows remained in the same rank through the time period, which is contrary to expectation. The great variation is supported by Kendall’s coefficient of concordance (W=0.15), which shows a low concordance and thereby indicates a considerable variation in ranking across the days. In addition, the *p*-value from the Kendall’s coefficient of concordance (p<0.001) indicates that the low concordance is significant. Because the rank varies so widely, no pattern can be derived that allows us to deduce anything about the possible leader of the herd.

The expected pattern of distribution of rank in this study is supported by Kondo et al. [25], who investigated the spatial and social behavior in a herd of beef cattle on grazing pasture and in a dry-lot. This study showed that changes in the surroundings had no influence on individuals who were assigned overall low or high rank. This may indicate that the roles of leaders and followers are quite stable, which is supported by the results in Arave and Albright [19], suggesting social status can be stable over a period of several years in an established herd. Additionally, Tibbetts et al. [38] state that hierarchies are commonly maintained over long periods of time. This is due to individuals saving time and energy, while also minimizing the risk of injuries by respecting an established hierarchy [38]. Unfortunately, no information about the distances between the different individuals and their leadership could be found in the international scientific literature. Greater contextualizing of the results is, therefore, not possible in this study.

The density plots indicate that the cattle generally have more ranks centered around the middle values and rarely have high and low ranks. This can be seen in the rank values ranging from 3 to 16, and with the graph peak values lying between 6 and 11. This is also supported by the boxplots, where the medians for each individual are relatively close to each other, with values between 7 and 10. Despite the low concordance shown in the Kendall’s coefficient of concordance, few of the density plots indicate skewness and thereby a tendency to either higher or lower ranks. Calculating the skewness confirms that some cows are right-skewed, while no clear left-skewness is shown for any of the cattle. The kurtosis of the density plots shows several values close to 3, which indicates that a lot of the values are centered around the mean. Therefore, the cattle fall between the middle ranks. There are also a few individuals who show a kurtosis value above 3, which indicates that their distribution is peaked. This suggests more variation in the reported ranks. Individuals showing a high kurtosis also show an extreme right-skewness, indicating a tendency of having higher ranks. This is supported by the moderate correlation between the peak x-values and the skewness (ρ=−0.53). The cumulative graph shows some clustering around the end. This might indicate an underlying pattern in the computed rank that could indicate leadership and follower roles. The cluster with the lowest cumulative rank, indicating the highest rank throughout the days, consists of Cow01, Cow05, and Cow06. When comparing results of the cumulative graph to the other analyses conducted in this study, it is still unclear if this pattern is conclusive. A possible improvement would be to extend the study period as the clustering might become more visible and clear in the other analyses.

Interestingly, age did not seem to have an impact on the results. Age is a factor known to potentially affect the hierarchy within a herd of cattle [19]. It would be plausible to assume that the sole cow (Cow08) in a herd of heifers would have a higher rank. However, this assumption is not supported by the findings of this study. Cow08 generally varied in observed ranks with a peak x-value of 8.88 in the density plot, indicating a tendency to rank around the middle. Furthermore, in the cumulative rank analysis, Cow08 was found to have the third highest cumulative rank. These observations suggest that the age of the individual did not have an impact on the findings of this study. Additionally, the relatively young age of the rest of the herd is noteworthy. Younger cattle tend to challenge each other and the established social composition, which may result in instability in hierarchical positions [19,39]. This might explain some of the great variation seen in this study. The older cow’s presence might have introduced unique social dynamics, but the younger heifers’ tendencies to frequently challenge social positions likely overshadowed any potential age-based influence. Future research should consider the potential influence of such outliers in the herd composition, as older cows may have different social influences compared to younger heifers.

Though this study shows tendencies suggesting a leadership pattern, no clear pattern can be extracted when looking at the data collectively. A pattern in the hierarchy and spatial distribution was also absent in Gabrieli and Malkinson [40]. They examined a herd of 130 cows by monitoring agonistic behavior, measuring distances between the cows during rest and grazing, and calculating their fitness, to find a correlation between dominance and fitness. They did not find a pattern in dominant behavior, and concluded that no cow consistently ranked first, middle, or last. Furthermore, no pattern could be found in individual positioning [40]. This is consistent with the results of this study. The lack of consistency in the spatial distribution of the cattle in this study may be due to the median-based method, which may not be optimal to examine spatial distribution.

### 4.3. Method Considerations

The method in this study uses median values, meaning a cow is less likely to have an overall low or high rank. The expected patterns of few individuals with high ranks and few individuals with low ranks were, therefore, not seen. Another way to process the data could be by using the sum, mean, or geometric mean instead of medians. However, this requires no loss of signal in the data collection. Since this system has several periods without data, it was not possible to use these methods in this study. Another way to improve the method could be by increasing the message frequency. In this study, a message was given for every 15 min. However, the messages were not aligned between individuals, so we used the median. While the improvement of signals might enhance the method in this study, other methods might also be important to take into consideration. Noteworthily, the cumulative ranks curves show a clearer pattern with three distinct groups in calculated ranks: low rank, middle rank and high rank (Figure 4). This suggests that in order to discover the leading cows (which could bear the collars alone), it is necessary to estimate the hierarchical position over multiple days in order to reduce the effect of the inter-day fluctuations in hierarchical positions. Technological advancements could possibly enhance the accuracy and applicability of the method used in this study. Improvements in GPS accuracy and signal reliability would reduce data loss and enable more precise tracking of individual positions. Furthermore, advancements in the collar designs, like enhanced battery life and connectivity, could further support continuous data collection, making virtual fencing systems more robust.

### 4.4. Alternative Methods

According to Tzanidakis et al. [18], the first step in making a model to study the behavior of livestock, is to make observations. It might be possible to prevent the signal failure issues in this study, or similar GPS technology studies, through physical observation. Multiple studies use physical observation to calculate a win:loss ratio and use this as a dominance value instead of only basing it on distances [19,25,40]. The win:loss ratio is based on observed agonistic encounters, where the number of interactions (such as threats and pushing) won is compared to the number of interactions lost. The cattle are then ranked by dominance based on these values [25,40]. Physical observations also have some disadvantages, as they are time-consuming, thus demanding additional labor and financial resources [41]. Additionally, physical observations may be more inaccurate due to the risk of missing important cues [42]. Instead of using physical observations to investigate behavior in cattle, Tzanidakis et al. [18] suggest that GPS-GIS technology could be used in future studies. In order to conduct a comprehensive analysis of cattle social dynamics within a virtual fence system, it may be necessary to combine several of these methods so the system can be implemented for agricultural use as well as in rewilding projects. In this study, only the physical positions of individuals in relation to the herd were taken into account. Including the use of the distribution of the individuals in relation to each other might have been able to reveal patterns between individuals, thereby finding a pattern within the herd. By including the distribution, it might be plausible to include the complexity of social roles and leadership. Understanding these spatial and social dynamics has practical applications for both farmers and conservationists. This can help farmers reduce the number of collars needed, lowering costs, and aiding conservationists in managing large-scale rewilding projects.

The limitations of this study are mainly due to the complexity of herd behavior and leadership. While this study aimed to simplify this with the use of distances in a virtual fences system, it proved to be a challenging task. The uncertainty of the data collected and the use of a median-based method limits this study. However, this way of interpreting herd behavior still has potential, as seen in the cumulative curves.

## 5. Conclusions

In conclusion, this study shows no clear pattern of leadership in a virtual fence system, when only investigating distances between individual herd members. While some tendencies could be discerned, only a low concordance between the days could be found. The method used in this study may have been affected by timing differences between collar messages, requiring us to use median values, which might be inadequate to describe herd positioning. Future studies could consider the distribution of the herd in relation to each other, thereby incorporating individual relationships and not just analyzing the herd as one unit. Another possibility is to extend the study period as clearer indication of a pattern might arise. As a result of this study, it has not been possible to identify a pattern that would allow only a limited number of collars to be applied to a specific number of cows. More studies are necessary to investigate whether it is possible to reduce the expenses of a virtual fence system and make it more applicable for farming on a large-scale and in rewilding projects. With continued research and development, virtual fencing could become a cost-effective and widely applicable tool in modern agricultural practice.

## Figures and Tables

**Table 1:** Skewness, kurtosis, and peak values, calculated and extracted from Figure 5.

	Skewness	Kurtosis	Peak x-Value	Peak y-Value
			(Rank)	(%)
Cow01	1.33	5.09	6.12	24.22
Cow02	0.31	2.22	8.73	19.67
Cow03	0.25	2.97	7.55	23.33
Cow04	−0.29	2.44	8.23	17.25
Cow05	0.09	2.07	6.50	16.07
Cow06	1.07	5.09	6.86	24.81
Cow07	0.67	2.56	6.84	20.35
Cow08	0.57	2.77	8.88	21.86
Cow09	−0.33	2.33	10.30	17.86
Cow10	0.27	2.46	8.44	19.50
Cow11	−0.21	2.16	10.30	19.50
Cow12	0.68	3.92	8.66	23.13
Cow13	−0.05	2.95	8.51	18.35
Cow14	0.58	2.60	7.55	17.33
Cow15	0.99	4.53	8.13	18.78
Cow16	−0.12	1.91	10.71	16.34
Cow17	0.30	2.92	8.55	19.18

**Figure A1:**
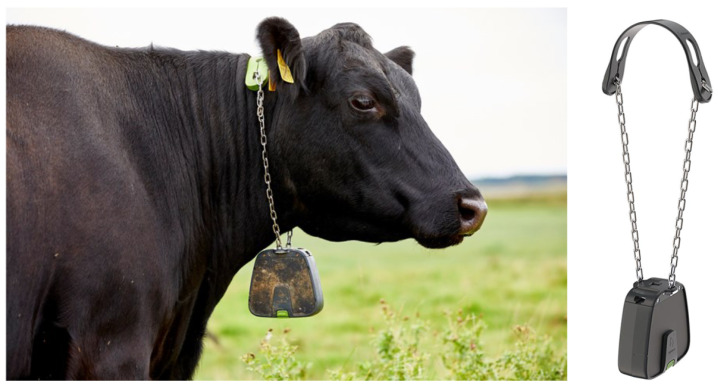
Nofence collar (Photo: J. Frikke).

**Figure A2:**
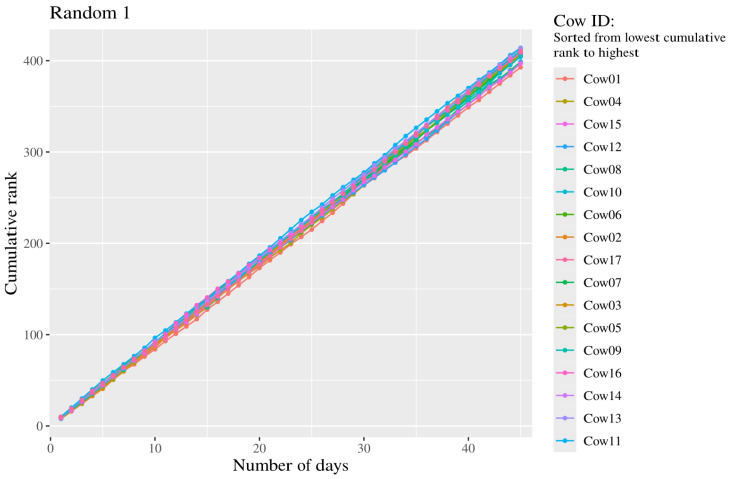

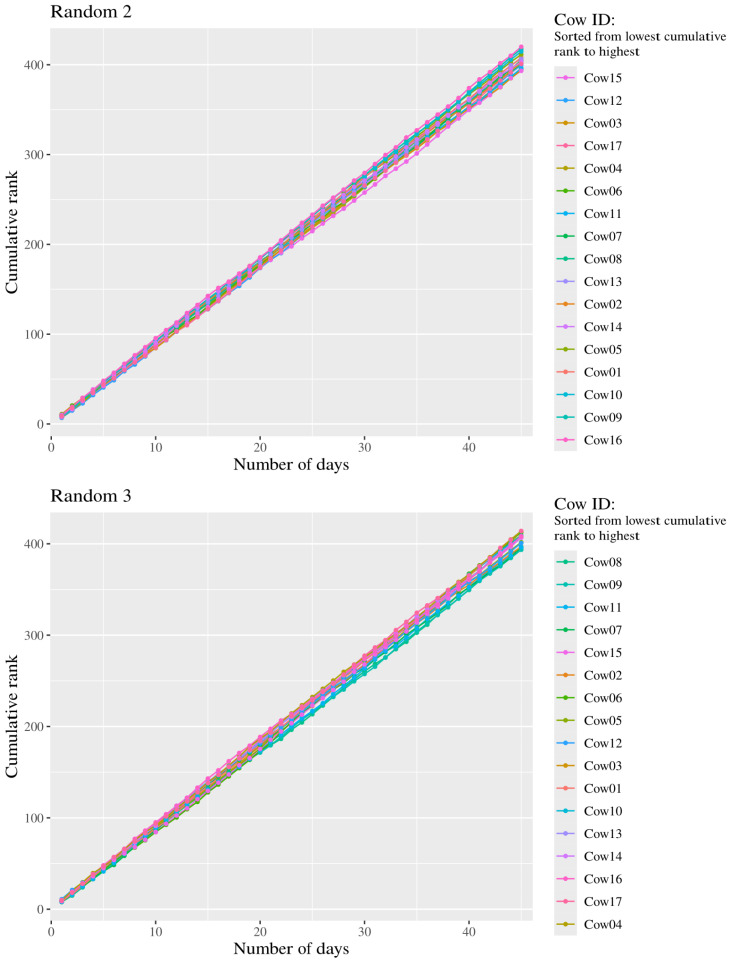

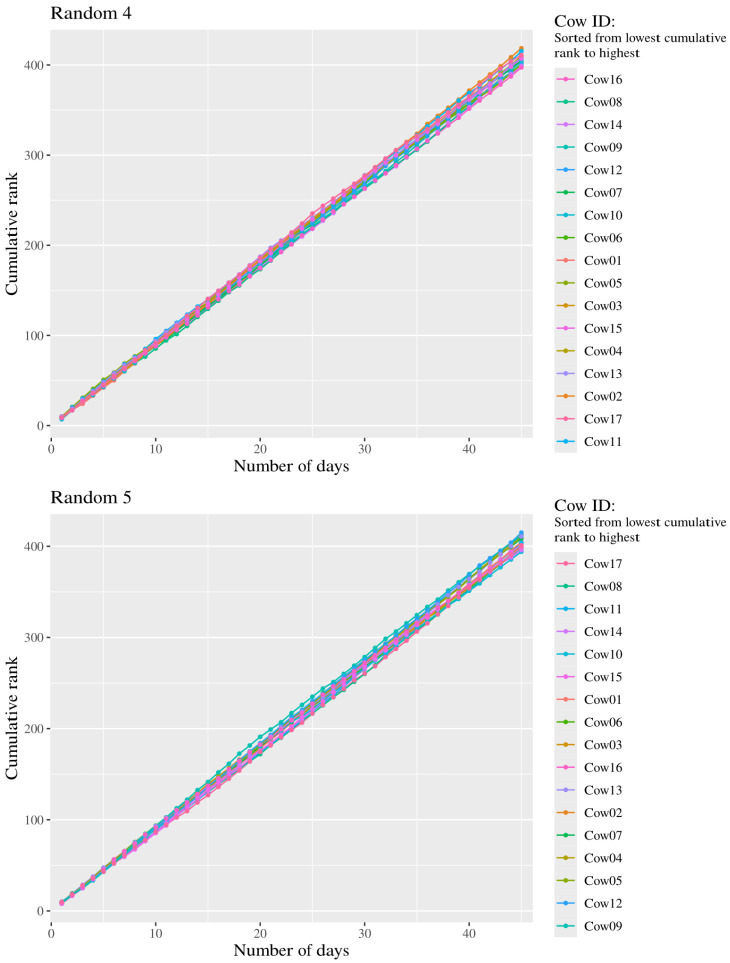

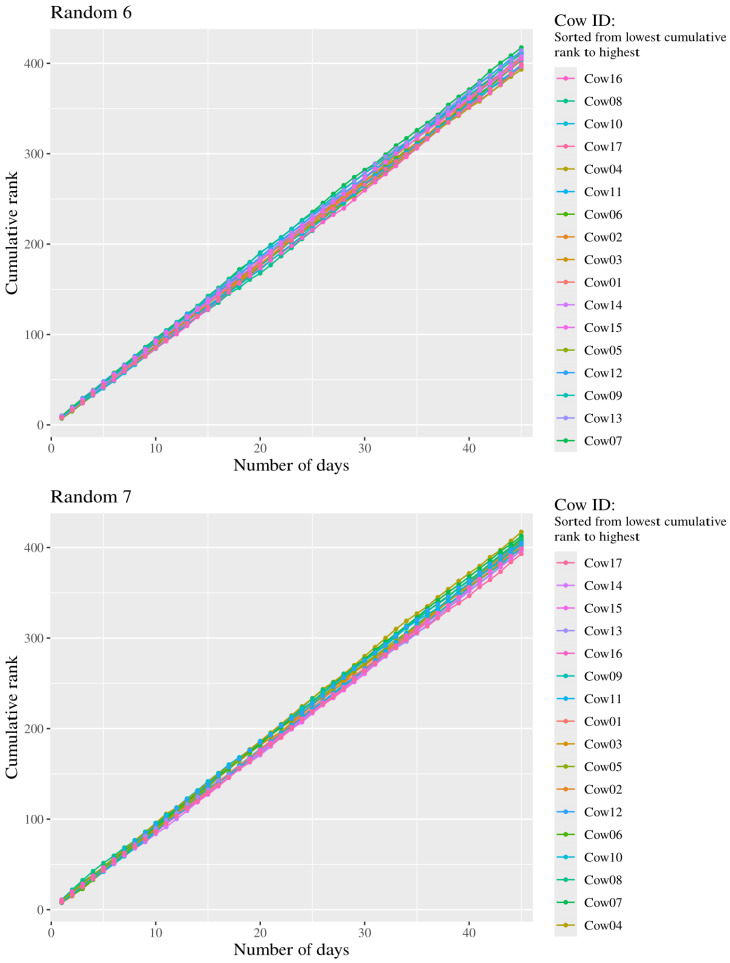

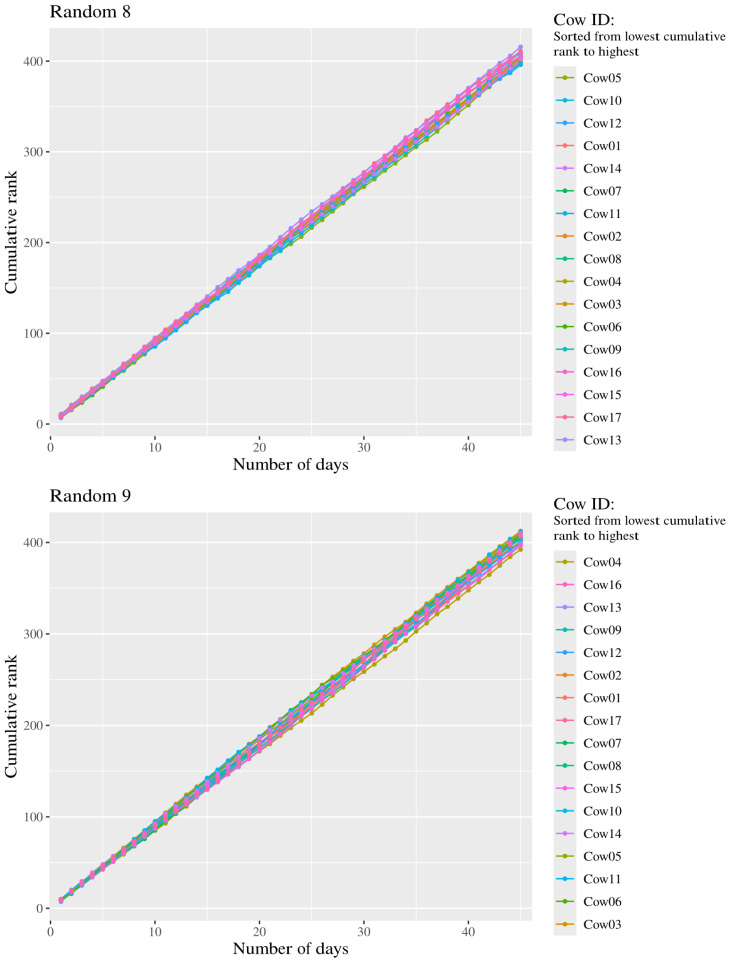

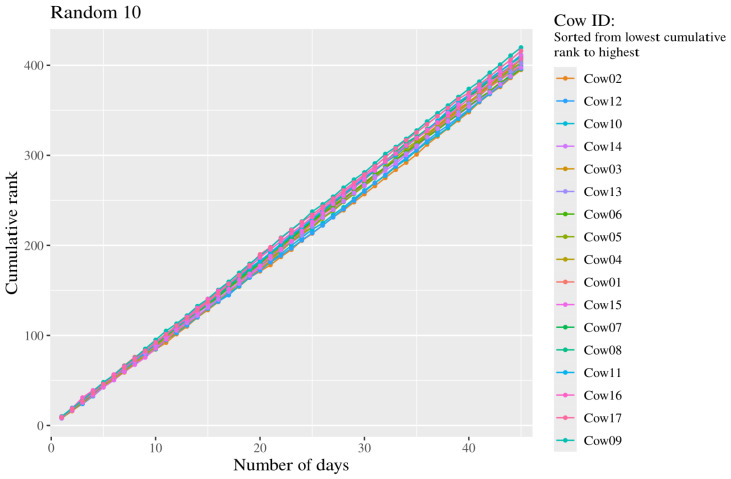
Cumulative ranks for each cow derived from the randomized data. Each color represents an individual in the herd. The Cow ID is sorted in ascending order from lowest to highest cumulative rank. A low cumulative rank indicates an overall high rank.

**Figure A3:**
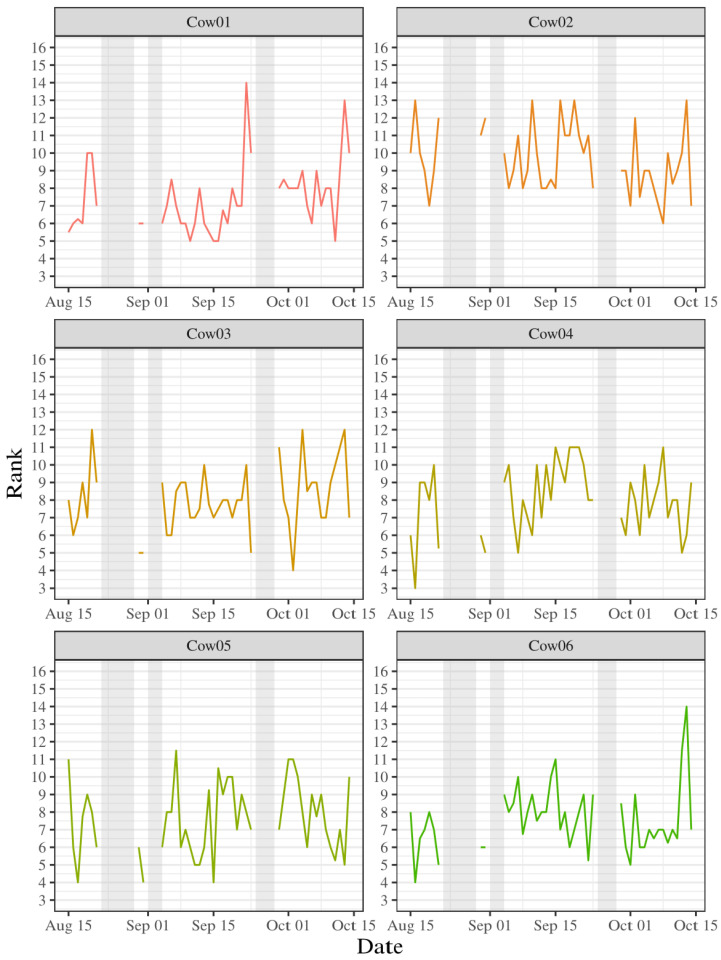

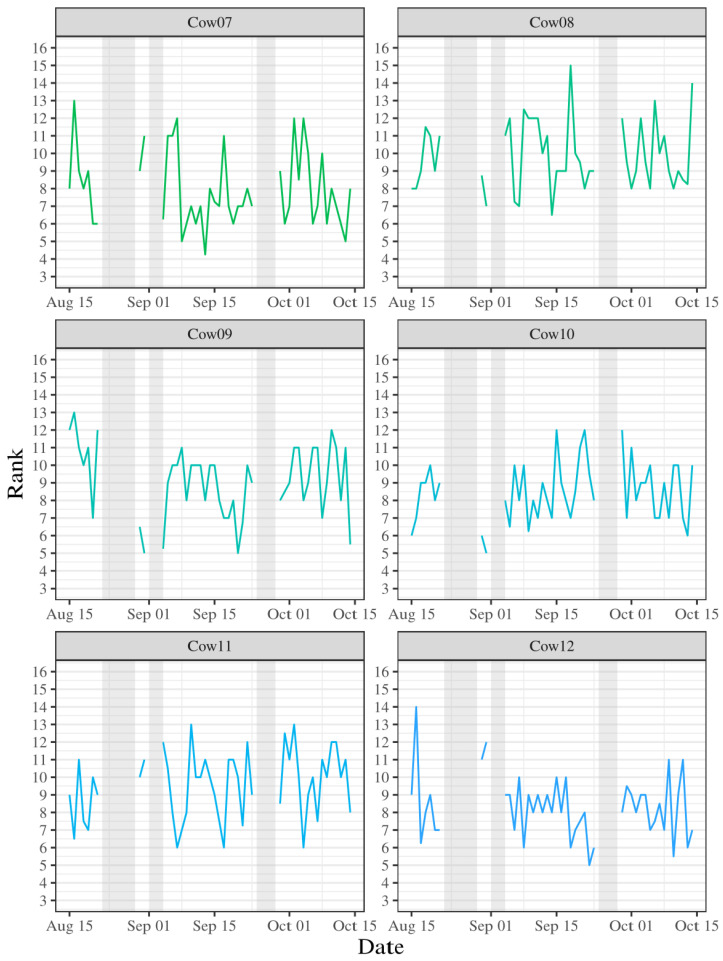

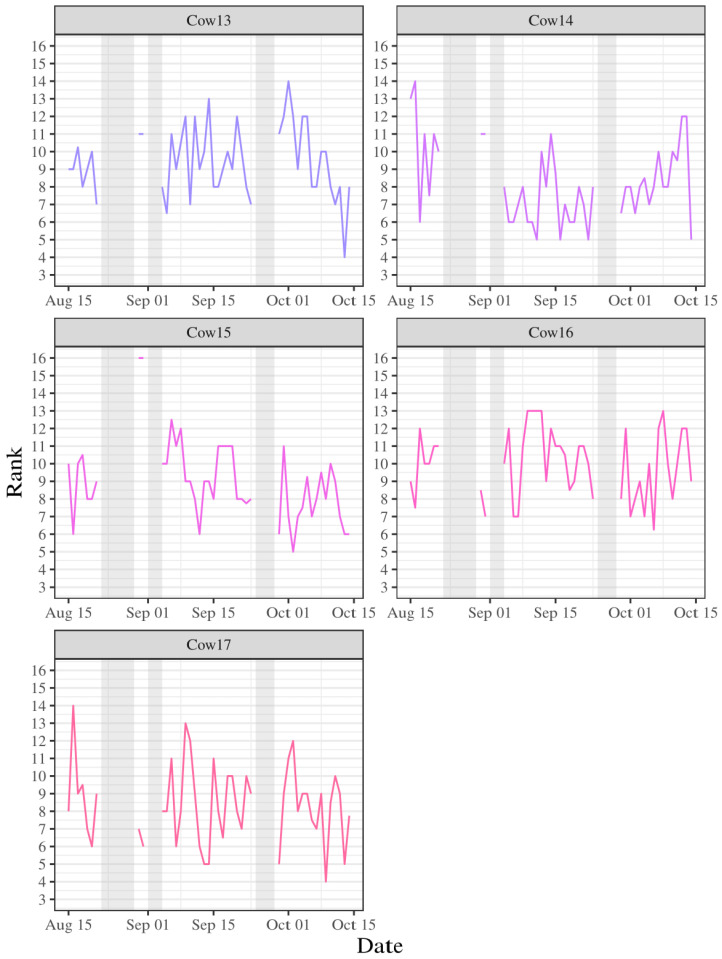
Times series of ranks based on medians within the herd throughout the 45-day study period. Each color represents each individual cow in the herd. The period of study started on 15 August and was concluded on the 15 October. The grey marked areas show the excluded dates, resulting from signal issues.

**Figure A4:**
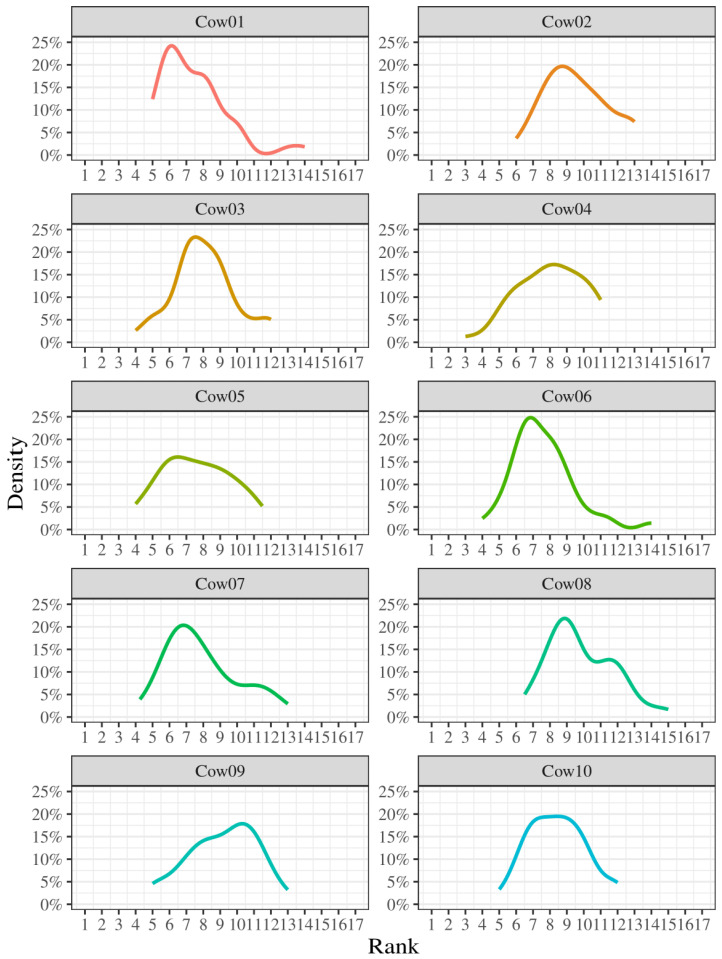

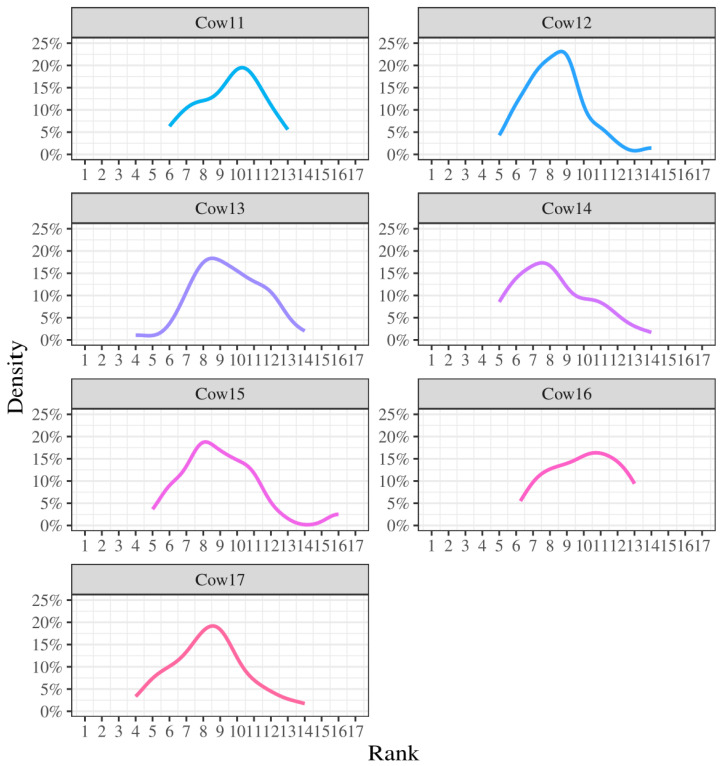
Density plot of ranks based on median values for each cow during the 45-day study period. Density is represented with percentages illustrating the amount of times an individual is observed in a specific rank. Each color represents an individual in the herd.

## Data Availability

The data presented in this study are available on request from the corresponding author.

## Appendix B

**Table A1:** The serial number from the Nofence© collars, and their reference name in this study. One collar lost connection and was replaced after three days, which resulted in Cow01 having two serial numbers.

Reference	Collar Serial Number
Cow01	73798
	90894
Cow02	73882
Cow03	73957
Cow04	73985
Cow05	74042
Cow06	82461
Cow07	82463
Cow08	82513
Cow09	82573
Cow10	82587
Cow11	84878
Cow12	89446
Cow13	89481
Cow14	89495
Cow15	90747
Cow16	90754
Cow17	90876

## Appendix C

**Table A2:** An overview of the number of observations of each cow used to find distances. Contains poll messages, client warning, and client zap.

	Number of Observations
Cow01	4212
Cow02	4272
Cow03	4187
Cow04	4276
Cow05	4238
Cow06	4291
Cow07	4340
Cow08	4382
Cow09	4297
Cow10	4706
Cow11	4219
Cow12	4308
Cow13	4314
Cow14	4193
Cow15	4057
Cow16	4180
Cow17	4297
Total	72,769

## Appendix E

**Table A3:** *p*-values of all the randomized medians of the hierarchical positions between all the cows with Mann–Whitney U test.

Pair	*p*-Value
Sample 1–Sample 2	0.628
Sample 1–Sample 3	0.468
Sample 1–Sample 4	0.814
Sample 1–Sample 5	0.533
Sample 1–Sample 6	0.978
Sample 1–Sample 7	0.440
Sample 1–Sample 8	0.570
Sample 1–Sample 9	0.436
Sample 1–Sample 10	0.690
Sample 2–Sample 3	0.813
Sample 2–Sample 4	0.800
Sample 2–Sample 5	0.900
Sample 2–Sample 6	0.656
Sample 2–Sample 7	0.783
Sample 2–Sample 8	0.942
Sample 2–Sample 9	0.785
Sample 2–Sample 10	0.929
Sample 3–Sample 4	0.606
Sample 3–Sample 5	0.886
Sample 3–Sample 6	0.478
Sample 3–Sample 7	0.958
Sample 3–Sample 8	0.849
Sample 3–Sample 9	0.997
Sample 3–Sample 10	0.722
Sample 4–Sample 5	0.715
Sample 4–Sample 6	0.812
Sample 4–Sample 7	0.591
Sample 4–Sample 8	0.749
Sample 4–Sample 9	0.599
Sample 4–Sample 10	0.878
Sample 5–Sample 6	0.543
Sample 5–Sample 7	0.864
Sample 5–Sample 8	0.964
Sample 5–Sample 9	0.857
Sample 5–Sample 10	0.834
Sample 6–Sample 7	0.475
Sample 6–Sample 8	0.579
Sample 6–Sample 9	0.436
Sample 6–Sample 10	0.690
Sample 7–Sample 8	0.819
Sample 7–Sample 9	0.975
Sample 7–Sample 10	0.715
Sample 8–Sample 9	0.827
Sample 8–Sample 10	0.869
Sample 9–Sample 10	0.704

## Appendix F

**Table A4:** *p*-values of the original data set compared to all the randomized medians of the hierarchical positions between all the cows with Mann–Whitney U test.

Pair	*p*-Value
Original–Sample 1	$4.763\times 10^{-9}$
Original–Sample 2	$5.070\times 10^{-8}$
Original–Sample 3	$5.793\times 10^{-8}$
Original–Sample 4	$7.327\times 10^{-9}$
Original–Sample 5	$2.726\times 10^{-8}$
Original–Sample 6	$1.197\times 10^{-8}$
Original–Sample 7	$1.177\times 10^{-7}$
Original–Sample 8	$2.031\times 10^{-8}$
Original–Sample 9	$2.530\times 10^{-8}$
Original–Sample 10	$1.769\times 10^{-8}$

## Appendix G

**Table A5:** Matrices of Man–Whitney U test between the individual cows before and after randomization of distances. Green indicates significant differences (*p*-values below 0.05), while red is insignificant.

**Original data (non-randomised)**																	
	**Cow01**	**Cow02**	**Cow03**	**Cow04**	**Cow05**	**Cow06**	**Cow07**	**Cow08**	**Cow09**	**Cow10**	**Cow11**	**Cow12**	**Cow13**	**Cow14**	**Cow15**	**Cow16**	**Cow17**
**Cow01**	NA	$4.20\times 10^{-7}$	$4.30\times 10^{-2}$	$5.50\times 10^{-2}$	$5.10\times 10^{-1}$	$4.50\times 10^{-1}$	$2.10\times 10^{-1}$	$6.00\times 10^{-8}$	$7.50\times 10^{-5}$	$2.10\times 10^{-3}$	$1.10\times 10^{-6}$	$6.70\times 10^{-3}$	$1.70\times 10^{-6}$	$7.20\times 10^{-2}$	$3.10\times 10^{-4}$	$5.50\times 10^{-8}$	$1.90\times 10^{-2}$
**Cow02**	$4.20\times 10^{-7}$	NA	$2.80\times 10^{-4}$	$9.30\times 10^{-4}$	$3.60\times 10^{-5}$	$1.20\times 10^{-6}$	$1.20\times 10^{-4}$	$6.30\times 10^{-1}$	$3.80\times 10^{-1}$	$6.30\times 10^{-3}$	$9.10\times 10^{-1}$	$1.90\times 10^{-3}$	$8.20\times 10^{-1}$	$1.70\times 10^{-3}$	$1.10\times 10^{-1}$	$3.40\times 10^{-1}$	$7.50\times 10^{-3}$
**Cow03**	$4.30\times 10^{-2}$	$2.80\times 10^{-4}$	NA	$8.70\times 10^{-1}$	$3.00\times 10^{-1}$	$1.10\times 10^{-1}$	$4.40\times 10^{-1}$	$4.10\times 10^{-5}$	$1.20\times 10^{-2}$	$3.00\times 10^{-1}$	$4.70\times 10^{-4}$	$4.90\times 10^{-1}$	$8.10\times 10^{-4}$	$9.70\times 10^{-1}$	$4.80\times 10^{-2}$	$3.10\times 10^{-5}$	$4.50\times 10^{-1}$
**Cow04**	$5.50\times 10^{-2}$	$9.30\times 10^{-4}$	$8.70\times 10^{-1}$	NA	$2.90\times 10^{-1}$	$1.50\times 10^{-1}$	$5.70\times 10^{-1}$	$1.80\times 10^{-4}$	$1.60\times 10^{-2}$	$4.00\times 10^{-1}$	$7.10\times 10^{-4}$	$6.70\times 10^{-1}$	$1.90\times 10^{-3}$	$9.50\times 10^{-1}$	$8.20\times 10^{-2}$	$4.30\times 10^{-5}$	$6.30\times 10^{-1}$
**Cow05**	$5.10\times 10^{-1}$	$3.60\times 10^{-5}$	$3.00\times 10^{-1}$	$2.90\times 10^{-1}$	NA	$9.00\times 10^{-1}$	$5.50\times 10^{-1}$	$6.00\times 10^{-6}$	$1.30\times 10^{-3}$	$4.40\times 10^{-2}$	$3.80\times 10^{-5}$	$1.10\times 10^{-1}$	$8.20\times 10^{-5}$	$2.40\times 10^{-1}$	$6.10\times 10^{-3}$	$1.90\times 10^{-6}$	$1.30\times 10^{-1}$
**Cow06**	$4.50\times 10^{-1}$	$1.20\times 10^{-6}$	$1.10\times 10^{-1}$	$1.50\times 10^{-1}$	$9.00\times 10^{-1}$	NA	$5.70\times 10^{-1}$	$1.10\times 10^{-7}$	$2.20\times 10^{-4}$	$9.50\times 10^{-3}$	$4.50\times 10^{-6}$	$2.50\times 10^{-2}$	$4.40\times 10^{-6}$	$2.10\times 10^{-1}$	$1.20\times 10^{-3}$	$1.60\times 10^{-7}$	$4.60\times 10^{-2}$
**Cow07**	$2.10\times 10^{-1}$	$1.20\times 10^{-4}$	$4.40\times 10^{-1}$	$5.70\times 10^{-1}$	$5.50\times 10^{-1}$	$5.70\times 10^{-1}$	NA	$2.00\times 10^{-5}$	$8.00\times 10^{-3}$	$1.00\times 10^{-1}$	$3.30\times 10^{-4}$	$1.80\times 10^{-1}$	$3.60\times 10^{-4}$	$5.30\times 10^{-1}$	$2.00\times 10^{-2}$	$1.70\times 10^{-5}$	$2.40\times 10^{-1}$
**Cow08**	$6.00\times 10^{-8}$	$6.30\times 10^{-1}$	$4.10\times 10^{-5}$	$1.80\times 10^{-4}$	$6.00\times 10^{-6}$	$1.10\times 10^{-7}$	$2.00\times 10^{-5}$	NA	$1.80\times 10^{-1}$	$1.60\times 10^{-3}$	$7.80\times 10^{-1}$	$3.20\times 10^{-4}$	$4.70\times 10^{-1}$	$3.10\times 10^{-4}$	$4.00\times 10^{-2}$	$6.10\times 10^{-1}$	$1.90\times 10^{-3}$
**Cow09**	$7.50\times 10^{-5}$	$3.80\times 10^{-1}$	$1.20\times 10^{-2}$	$1.60\times 10^{-2}$	$1.30\times 10^{-3}$	$2.20\times 10^{-4}$	$8.00\times 10^{-3}$	$1.80\times 10^{-1}$	NA	$8.30\times 10^{-2}$	$3.20\times 10^{-1}$	$3.60\times 10^{-2}$	$4.90\times 10^{-1}$	$3.80\times 10^{-2}$	$5.00\times 10^{-1}$	$5.80\times 10^{-2}$	$7.10\times 10^{-2}$
**Cow10**	$2.10\times 10^{-3}$	$6.30\times 10^{-3}$	$3.00\times 10^{-1}$	$4.00\times 10^{-1}$	$4.40\times 10^{-2}$	$9.50\times 10^{-3}$	$1.00\times 10^{-1}$	$1.60\times 10^{-3}$	$8.30\times 10^{-2}$	NA	$5.20\times 10^{-3}$	$6.50\times 10^{-1}$	$1.30\times 10^{-2}$	$3.80\times 10^{-1}$	$3.20\times 10^{-1}$	$4.20\times 10^{-4}$	$7.70\times 10^{-1}$
**Cow11**	$1.10\times 10^{-6}$	$9.10\times 10^{-1}$	$4.70\times 10^{-4}$	$7.10\times 10^{-4}$	$3.80\times 10^{-5}$	$4.50\times 10^{-6}$	$3.30\times 10^{-4}$	$7.80\times 10^{-1}$	$3.20\times 10^{-1}$	$5.20\times 10^{-3}$	NA	$1.70\times 10^{-3}$	$7.80\times 10^{-1}$	$3.60\times 10^{-3}$	$1.00\times 10^{-1}$	$3.50\times 10^{-1}$	$6.50\times 10^{-3}$
**Cow12**	$6.70\times 10^{-3}$	$1.90\times 10^{-3}$	$4.90\times 10^{-1}$	$6.70\times 10^{-1}$	$1.10\times 10^{-1}$	$2.50\times 10^{-2}$	$1.80\times 10^{-1}$	$3.20\times 10^{-4}$	$3.60\times 10^{-2}$	$6.50\times 10^{-1}$	$1.70\times 10^{-3}$	NA	$4.30\times 10^{-3}$	$5.80\times 10^{-1}$	$1.50\times 10^{-1}$	$1.50\times 10^{-4}$	$9.10\times 10^{-1}$
**Cow13**	$1.70\times 10^{-6}$	$8.20\times 10^{-1}$	$8.10\times 10^{-4}$	$1.90\times 10^{-3}$	$8.20\times 10^{-5}$	$4.40\times 10^{-6}$	$3.60\times 10^{-4}$	$4.70\times 10^{-1}$	$4.90\times 10^{-1}$	$1.30\times 10^{-2}$	$7.80\times 10^{-1}$	$4.30\times 10^{-3}$	NA	$3.90\times 10^{-3}$	$1.70\times 10^{-1}$	$2.50\times 10^{-1}$	$1.50\times 10^{-2}$
**Cow14**	$7.20\times 10^{-2}$	$1.70\times 10^{-3}$	$9.70\times 10^{-1}$	$9.50\times 10^{-1}$	$2.40\times 10^{-1}$	$2.10\times 10^{-1}$	$5.30\times 10^{-1}$	$3.10\times 10^{-4}$	$3.80\times 10^{-2}$	$3.80\times 10^{-1}$	$3.60\times 10^{-3}$	$5.80\times 10^{-1}$	$3.90\times 10^{-3}$	NA	$1.10\times 10^{-1}$	$2.30\times 10^{-4}$	$6.20\times 10^{-1}$
**Cow15**	$3.10\times 10^{-4}$	$1.10\times 10^{-1}$	$4.80\times 10^{-2}$	$8.20\times 10^{-2}$	$6.10\times 10^{-3}$	$1.20\times 10^{-3}$	$2.00\times 10^{-2}$	$4.00\times 10^{-2}$	$5.00\times 10^{-1}$	$3.20\times 10^{-1}$	$1.00\times 10^{-1}$	$1.50\times 10^{-1}$	$1.70\times 10^{-1}$	$1.10\times 10^{-1}$	NA	$1.60\times 10^{-2}$	$2.40\times 10^{-1}$
**Cow16**	$5.50\times 10^{-8}$	$3.40\times 10^{-1}$	$3.10\times 10^{-5}$	$4.30\times 10^{-5}$	$1.90\times 10^{-6}$	$1.60\times 10^{-7}$	$1.70\times 10^{-5}$	$6.10\times 10^{-1}$	$5.80\times 10^{-2}$	$4.20\times 10^{-4}$	$3.50\times 10^{-1}$	$1.50\times 10^{-4}$	$2.50\times 10^{-1}$	$2.30\times 10^{-4}$	$1.60\times 10^{-2}$	NA	$7.40\times 10^{-4}$
**Cow17**	$1.90\times 10^{-2}$	$7.50\times 10^{-3}$	$4.50\times 10^{-1}$	$6.30\times 10^{-1}$	$1.30\times 10^{-1}$	$4.60\times 10^{-2}$	$2.40\times 10^{-1}$	$1.90\times 10^{-3}$	$7.10\times 10^{-2}$	$7.70\times 10^{-1}$	$6.50\times 10^{-3}$	$9.10\times 10^{-1}$	$1.50\times 10^{-2}$	$6.20\times 10^{-1}$	$2.40\times 10^{-1}$	$7.40\times 10^{-4}$	NA
**Randomized data 1**																	
	**Cow01**	**Cow02**	**Cow03**	**Cow04**	**Cow05**	**Cow06**	**Cow07**	**Cow08**	**Cow09**	**Cow10**	**Cow11**	**Cow12**	**Cow13**	**Cow14**	**Cow15**	**Cow16**	**Cow17**
**Cow01**	NA	$1.27\times 10^{-2}$	$4.59\times 10^{-2}$	$5.45\times 10^{-1}$	$4.32\times 10^{-2}$	$1.66\times 10^{-2}$	$1.18\times 10^{-1}$	$4.72\times 10^{-1}$	$5.99\times 10^{-3}$	$1.19\times 10^{-1}$	$6.29\times 10^{-3}$	$3.58\times 10^{-1}$	$1.80\times 10^{-3}$	$8.87\times 10^{-3}$	$5.29\times 10^{-1}$	$2.31\times 10^{-2}$	$1.83\times 10^{-2}$
**Cow02**	$1.27\times 10^{-2}$	NA	$6.17\times 10^{-1}$	$5.15\times 10^{-2}$	$4.47\times 10^{-1}$	$6.25\times 10^{-1}$	$4.39\times 10^{-1}$	$1.42\times 10^{-1}$	$8.45\times 10^{-1}$	$3.09\times 10^{-1}$	$1.00\times 10^{0}$	$8.04\times 10^{-2}$	$9.97\times 10^{-1}$	$5.77\times 10^{-1}$	$8.25\times 10^{-2}$	$4.86\times 10^{-1}$	$8.78\times 10^{-1}$
**Cow03**	$4.59\times 10^{-2}$	$6.17\times 10^{-1}$	NA	$1.62\times 10^{-1}$	$9.08\times 10^{-1}$	$8.78\times 10^{-1}$	$7.14\times 10^{-1}$	$3.16\times 10^{-1}$	$6.73\times 10^{-1}$	$6.15\times 10^{-1}$	$5.93\times 10^{-1}$	$2.27\times 10^{-1}$	$5.33\times 10^{-1}$	$8.95\times 10^{-1}$	$2.32\times 10^{-1}$	$9.90\times 10^{-1}$	$7.44\times 10^{-1}$
**Cow04**	$5.45\times 10^{-1}$	$5.15\times 10^{-2}$	$1.62\times 10^{-1}$	NA	$1.70\times 10^{-1}$	$9.10\times 10^{-2}$	$2.97\times 10^{-1}$	$8.11\times 10^{-1}$	$4.27\times 10^{-2}$	$3.54\times 10^{-1}$	$3.54\times 10^{-2}$	$7.72\times 10^{-1}$	$1.91\times 10^{-2}$	$7.75\times 10^{-2}$	$9.18\times 10^{-1}$	$1.28\times 10^{-1}$	$7.75\times 10^{-2}$
**Cow05**	$4.32\times 10^{-2}$	$4.47\times 10^{-1}$	$9.08\times 10^{-1}$	$1.70\times 10^{-1}$	NA	$7.94\times 10^{-1}$	$8.66\times 10^{-1}$	$3.81\times 10^{-1}$	$5.84\times 10^{-1}$	$6.68\times 10^{-1}$	$4.93\times 10^{-1}$	$2.43\times 10^{-1}$	$4.47\times 10^{-1}$	$9.29\times 10^{-1}$	$2.83\times 10^{-1}$	$9.47\times 10^{-1}$	$6.10\times 10^{-1}$
**Cow06**	$1.66\times 10^{-2}$	$6.25\times 10^{-1}$	$8.78\times 10^{-1}$	$9.10\times 10^{-2}$	$7.94\times 10^{-1}$	NA	$5.94\times 10^{-1}$	$2.73\times 10^{-1}$	$7.99\times 10^{-1}$	$4.44\times 10^{-1}$	$6.88\times 10^{-1}$	$1.32\times 10^{-1}$	$6.81\times 10^{-1}$	$8.32\times 10^{-1}$	$2.05\times 10^{-1}$	$8.33\times 10^{-1}$	$8.10\times 10^{-1}$
**Cow07**	$1.18\times 10^{-1}$	$4.39\times 10^{-1}$	$7.14\times 10^{-1}$	$2.97\times 10^{-1}$	$8.66\times 10^{-1}$	$5.94\times 10^{-1}$	NA	$4.69\times 10^{-1}$	$3.97\times 10^{-1}$	$9.18\times 10^{-1}$	$3.29\times 10^{-1}$	$4.42\times 10^{-1}$	$2.78\times 10^{-1}$	$5.34\times 10^{-1}$	$3.52\times 10^{-1}$	$7.45\times 10^{-1}$	$4.73\times 10^{-1}$
**Cow08**	$4.72\times 10^{-1}$	$1.42\times 10^{-1}$	$3.16\times 10^{-1}$	$8.11\times 10^{-1}$	$3.81\times 10^{-1}$	$2.73\times 10^{-1}$	$4.69\times 10^{-1}$	NA	$1.64\times 10^{-1}$	$5.68\times 10^{-1}$	$1.28\times 10^{-1}$	$9.93\times 10^{-1}$	$1.02\times 10^{-1}$	$2.51\times 10^{-1}$	$8.68\times 10^{-1}$	$3.22\times 10^{-1}$	$2.00\times 10^{-1}$
**Cow09**	$5.99\times 10^{-3}$	$8.45\times 10^{-1}$	$6.73\times 10^{-1}$	$4.27\times 10^{-2}$	$5.84\times 10^{-1}$	$7.99\times 10^{-1}$	$3.97\times 10^{-1}$	$1.64\times 10^{-1}$	NA	$2.95\times 10^{-1}$	$8.99\times 10^{-1}$	$6.60\times 10^{-2}$	$8.89\times 10^{-1}$	$6.45\times 10^{-1}$	$1.12\times 10^{-1}$	$6.26\times 10^{-1}$	$9.90\times 10^{-1}$
**Cow10**	$1.19\times 10^{-1}$	$3.09\times 10^{-1}$	$6.15\times 10^{-1}$	$3.54\times 10^{-1}$	$6.68\times 10^{-1}$	$4.44\times 10^{-1}$	$9.18\times 10^{-1}$	$5.68\times 10^{-1}$	$2.95\times 10^{-1}$	NA	$2.69\times 10^{-1}$	$4.76\times 10^{-1}$	$1.96\times 10^{-1}$	$4.42\times 10^{-1}$	$4.68\times 10^{-1}$	$5.42\times 10^{-1}$	$3.81\times 10^{-1}$
**Cow11**	$6.29\times 10^{-3}$	$1.00\times 10^{0}$	$5.93\times 10^{-1}$	$3.54\times 10^{-2}$	$4.93\times 10^{-1}$	$6.88\times 10^{-1}$	$3.29\times 10^{-1}$	$1.28\times 10^{-1}$	$8.99\times 10^{-1}$	$2.69\times 10^{-1}$	NA	$6.25\times 10^{-2}$	$9.93\times 10^{-1}$	$6.22\times 10^{-1}$	$7.95\times 10^{-2}$	$5.33\times 10^{-1}$	$8.64\times 10^{-1}$
**Cow12**	$3.58\times 10^{-1}$	$8.04\times 10^{-2}$	$2.27\times 10^{-1}$	$7.72\times 10^{-1}$	$2.43\times 10^{-1}$	$1.32\times 10^{-1}$	$4.42\times 10^{-1}$	$9.93\times 10^{-1}$	$6.60\times 10^{-2}$	$4.76\times 10^{-1}$	$6.25\times 10^{-2}$	NA	$3.30\times 10^{-2}$	$1.10\times 10^{-1}$	$8.87\times 10^{-1}$	$1.79\times 10^{-1}$	$1.15\times 10^{-1}$
**Cow13**	$1.80\times 10^{-3}$	$9.97\times 10^{-1}$	$5.33\times 10^{-1}$	$1.91\times 10^{-2}$	$4.47\times 10^{-1}$	$6.81\times 10^{-1}$	$2.78\times 10^{-1}$	$1.02\times 10^{-1}$	$8.89\times 10^{-1}$	$1.96\times 10^{-1}$	$9.93\times 10^{-1}$	$3.30\times 10^{-2}$	NA	$5.17\times 10^{-1}$	$6.58\times 10^{-2}$	$4.73\times 10^{-1}$	$8.74\times 10^{-1}$
**Cow14**	$8.87\times 10^{-3}$	$5.77\times 10^{-1}$	$8.95\times 10^{-1}$	$7.75\times 10^{-2}$	$9.29\times 10^{-1}$	$8.32\times 10^{-1}$	$5.34\times 10^{-1}$	$2.51\times 10^{-1}$	$6.45\times 10^{-1}$	$4.42\times 10^{-1}$	$6.22\times 10^{-1}$	$1.10\times 10^{-1}$	$5.17\times 10^{-1}$	NA	$2.02\times 10^{-1}$	$9.86\times 10^{-1}$	$7.60\times 10^{-1}$
**Cow15**	$5.29\times 10^{-1}$	$8.25\times 10^{-2}$	$2.32\times 10^{-1}$	$9.18\times 10^{-1}$	$2.83\times 10^{-1}$	$2.05\times 10^{-1}$	$3.52\times 10^{-1}$	$8.68\times 10^{-1}$	$1.12\times 10^{-1}$	$4.68\times 10^{-1}$	$7.95\times 10^{-2}$	$8.87\times 10^{-1}$	$6.58\times 10^{-2}$	$2.02\times 10^{-1}$	NA	$2.59\times 10^{-1}$	$1.36\times 10^{-1}$
**Cow16**	$2.31\times 10^{-2}$	$4.86\times 10^{-1}$	$9.90\times 10^{-1}$	$1.28\times 10^{-1}$	$9.47\times 10^{-1}$	$8.33\times 10^{-1}$	$7.45\times 10^{-1}$	$3.22\times 10^{-1}$	$6.26\times 10^{-1}$	$5.42\times 10^{-1}$	$5.33\times 10^{-1}$	$1.79\times 10^{-1}$	$4.73\times 10^{-1}$	$9.86\times 10^{-1}$	$2.59\times 10^{-1}$	NA	$6.74\times 10^{-1}$
**Cow17**	$1.83\times 10^{-2}$	$8.78\times 10^{-1}$	$7.44\times 10^{-1}$	$7.75\times 10^{-2}$	$6.10\times 10^{-1}$	$8.10\times 10^{-1}$	$4.73\times 10^{-1}$	$2.00\times 10^{-1}$	$9.90\times 10^{-1}$	$3.81\times 10^{-1}$	$8.64\times 10^{-1}$	$1.15\times 10^{-1}$	$8.74\times 10^{-1}$	$7.60\times 10^{-1}$	$1.36\times 10^{-1}$	$6.74\times 10^{-1}$	NA
**Randomized data 2**																	
	**Cow01**	**Cow02**	**Cow03**	**Cow04**	**Cow05**	**Cow06**	**Cow07**	**Cow08**	**Cow09**	**Cow10**	**Cow11**	**Cow12**	**Cow13**	**Cow14**	**Cow15**	**Cow16**	**Cow17**
**Cow01**	NA	$4.94\times 10^{-1}$	$1.31\times 10^{-1}$	$1.82\times 10^{-1}$	$8.58\times 10^{-1}$	$1.00\times 10^{0}$	$5.69\times 10^{-1}$	$3.68\times 10^{-1}$	$2.73\times 10^{-1}$	$2.70\times 10^{-1}$	$1.20\times 10^{-1}$	$3.51\times 10^{-1}$	$9.63\times 10^{-1}$	$7.58\times 10^{-1}$	$2.90\times 10^{-2}$	$9.47\times 10^{-2}$	$3.67\times 10^{-1}$
**Cow02**	$4.94\times 10^{-1}$	NA	$3.83\times 10^{-1}$	$5.13\times 10^{-1}$	$3.98\times 10^{-1}$	$5.74\times 10^{-1}$	$9.01\times 10^{-1}$	$8.63\times 10^{-1}$	$1.07\times 10^{-1}$	$9.32\times 10^{-2}$	$3.40\times 10^{-1}$	$8.09\times 10^{-1}$	$6.35\times 10^{-1}$	$7.55\times 10^{-1}$	$1.51\times 10^{-1}$	$2.72\times 10^{-2}$	$8.73\times 10^{-1}$
**Cow03**	$1.31\times 10^{-1}$	$3.83\times 10^{-1}$	NA	$8.68\times 10^{-1}$	$9.01\times 10^{-2}$	$2.62\times 10^{-1}$	$3.56\times 10^{-1}$	$5.36\times 10^{-1}$	$2.02\times 10^{-2}$	$2.98\times 10^{-2}$	$9.97\times 10^{-1}$	$5.89\times 10^{-1}$	$2.05\times 10^{-1}$	$2.87\times 10^{-1}$	$8.20\times 10^{-1}$	$5.92\times 10^{-3}$	$4.55\times 10^{-1}$
**Cow04**	$1.82\times 10^{-1}$	$5.13\times 10^{-1}$	$8.68\times 10^{-1}$	NA	$1.30\times 10^{-1}$	$3.02\times 10^{-1}$	$4.53\times 10^{-1}$	$6.53\times 10^{-1}$	$2.85\times 10^{-2}$	$3.70\times 10^{-2}$	$8.48\times 10^{-1}$	$7.27\times 10^{-1}$	$2.76\times 10^{-1}$	$3.59\times 10^{-1}$	$6.01\times 10^{-1}$	$7.28\times 10^{-3}$	$6.11\times 10^{-1}$
**Cow05**	$8.58\times 10^{-1}$	$3.98\times 10^{-1}$	$9.01\times 10^{-2}$	$1.30\times 10^{-1}$	NA	$8.00\times 10^{-1}$	$4.30\times 10^{-1}$	$2.78\times 10^{-1}$	$4.06\times 10^{-1}$	$4.22\times 10^{-1}$	$7.61\times 10^{-2}$	$2.62\times 10^{-1}$	$8.22\times 10^{-1}$	$6.26\times 10^{-1}$	$2.50\times 10^{-2}$	$1.66\times 10^{-1}$	$3.15\times 10^{-1}$
**Cow06**	$1.00\times 10^{0}$	$5.74\times 10^{-1}$	$2.62\times 10^{-1}$	$3.02\times 10^{-1}$	$8.00\times 10^{-1}$	NA	$6.98\times 10^{-1}$	$4.95\times 10^{-1}$	$2.93\times 10^{-1}$	$3.94\times 10^{-1}$	$3.38\times 10^{-1}$	$4.51\times 10^{-1}$	$9.80\times 10^{-1}$	$7.94\times 10^{-1}$	$1.13\times 10^{-1}$	$1.66\times 10^{-1}$	$4.14\times 10^{-1}$
**Cow07**	$5.69\times 10^{-1}$	$9.01\times 10^{-1}$	$3.56\times 10^{-1}$	$4.53\times 10^{-1}$	$4.30\times 10^{-1}$	$6.98\times 10^{-1}$	NA	$7.82\times 10^{-1}$	$1.31\times 10^{-1}$	$1.39\times 10^{-1}$	$3.26\times 10^{-1}$	$6.96\times 10^{-1}$	$6.86\times 10^{-1}$	$8.34\times 10^{-1}$	$1.51\times 10^{-1}$	$3.58\times 10^{-2}$	$7.78\times 10^{-1}$
**Cow08**	$3.68\times 10^{-1}$	$8.63\times 10^{-1}$	$5.36\times 10^{-1}$	$6.53\times 10^{-1}$	$2.78\times 10^{-1}$	$4.95\times 10^{-1}$	$7.82\times 10^{-1}$	NA	$7.06\times 10^{-2}$	$8.09\times 10^{-2}$	$4.73\times 10^{-1}$	$9.11\times 10^{-1}$	$4.79\times 10^{-1}$	$5.90\times 10^{-1}$	$2.76\times 10^{-1}$	$1.64\times 10^{-2}$	$9.70\times 10^{-1}$
**Cow09**	$2.73\times 10^{-1}$	$1.07\times 10^{-1}$	$2.02\times 10^{-2}$	$2.85\times 10^{-2}$	$4.06\times 10^{-1}$	$2.93\times 10^{-1}$	$1.31\times 10^{-1}$	$7.06\times 10^{-2}$	NA	$9.06\times 10^{-1}$	$2.90\times 10^{-2}$	$7.04\times 10^{-2}$	$3.34\times 10^{-1}$	$1.76\times 10^{-1}$	$5.14\times 10^{-3}$	$7.34\times 10^{-1}$	$6.67\times 10^{-2}$
**Cow10**	$2.70\times 10^{-1}$	$9.32\times 10^{-2}$	$2.98\times 10^{-2}$	$3.70\times 10^{-2}$	$4.22\times 10^{-1}$	$3.94\times 10^{-1}$	$1.39\times 10^{-1}$	$8.09\times 10^{-2}$	$9.06\times 10^{-1}$	NA	$4.12\times 10^{-2}$	$7.17\times 10^{-2}$	$3.74\times 10^{-1}$	$2.17\times 10^{-1}$	$4.32\times 10^{-3}$	$5.77\times 10^{-1}$	$4.96\times 10^{-2}$
**Cow11**	$1.20\times 10^{-1}$	$3.40\times 10^{-1}$	$9.97\times 10^{0}$	$8.48\times 10^{-1}$	$7.61\times 10^{-2}$	$3.38\times 10^{-1}$	$3.26\times 10^{-1}$	$4.73\times 10^{-1}$	$2.90\times 10^{-2}$	$4.12\times 10^{-2}$	NA	$5.48\times 10^{-1}$	$2.26\times 10^{-1}$	$3.08\times 10^{-1}$	$9.87\times 10^{-1}$	$9.81\times 10^{-3}$	$3.57\times 10^{-1}$
**Cow12**	$3.51\times 10^{-1}$	$8.09\times 10^{-1}$	$5.89\times 10^{-1}$	$7.27\times 10^{-1}$	$2.62\times 10^{-1}$	$4.51\times 10^{-1}$	$6.96\times 10^{-1}$	$9.11\times 10^{-1}$	$7.04\times 10^{-2}$	$7.17\times 10^{-2}$	$5.48\times 10^{-1}$	NA	$4.78\times 10^{-1}$	$5.72\times 10^{-1}$	$3.11\times 10^{-1}$	$1.75\times 10^{-2}$	$9.19\times 10^{-1}$
**Cow13**	$9.63\times 10^{-1}$	$6.35\times 10^{-1}$	$2.05\times 10^{-1}$	$2.76\times 10^{-1}$	$8.22\times 10^{-1}$	$9.80\times 10^{-1}$	$6.86\times 10^{-1}$	$4.79\times 10^{-1}$	$3.34\times 10^{-1}$	$3.74\times 10^{-1}$	$2.26\times 10^{-1}$	$4.78\times 10^{-1}$	NA	$7.88\times 10^{-1}$	$1.00\times 10^{-1}$	$1.88\times 10^{-1}$	$5.21\times 10^{-1}$
**Cow14**	$7.58\times 10^{-1}$	$7.55\times 10^{-1}$	$2.87\times 10^{-1}$	$3.59\times 10^{-1}$	$6.26\times 10^{-1}$	$7.94\times 10^{-1}$	$8.34\times 10^{-1}$	$5.90\times 10^{-1}$	$1.76\times 10^{-1}$	$2.17\times 10^{-1}$	$3.08\times 10^{-1}$	$5.72\times 10^{-1}$	$7.88\times 10^{-1}$	NA	$1.18\times 10^{-1}$	$7.52\times 10^{-2}$	$6.05\times 10^{-1}$
**Cow15**	$2.90\times 10^{-2}$	$1.51\times 10^{-1}$	$8.20\times 10^{-1}$	$6.01\times 10^{-1}$	$2.50\times 10^{-2}$	$1.13\times 10^{-1}$	$1.51\times 10^{-1}$	$2.76\times 10^{-1}$	$5.14\times 10^{-3}$	$4.32\times 10^{-3}$	$9.87\times 10^{0}$	$3.11\times 10^{-1}$	$1.00\times 10^{-1}$	$1.18\times 10^{-1}$	NA	$6.91\times 10^{-4}$	$1.53\times 10^{-1}$
**Cow16**	$9.47\times 10^{-2}$	$2.72\times 10^{-2}$	$5.92\times 10^{-3}$	$7.28\times 10^{-3}$	$1.66\times 10^{-1}$	$1.66\times 10^{-1}$	$3.58\times 10^{-2}$	$1.64\times 10^{-2}$	$7.34\times 10^{-1}$	$5.77\times 10^{-1}$	$9.81\times 10^{-3}$	$1.75\times 10^{-2}$	$1.88\times 10^{-1}$	$7.52\times 10^{-2}$	$6.91\times 10^{-4}$	NA	$1.27\times 10^{-2}$
**Cow17**	$3.67\times 10^{-1}$	$8.73\times 10^{-1}$	$4.55\times 10^{-1}$	$6.11\times 10^{-1}$	$3.15\times 10^{-1}$	$4.14\times 10^{-1}$	$7.78\times 10^{-1}$	$9.70\times 10^{-1}$	$6.67\times 10^{-2}$	$4.96\times 10^{-2}$	$3.57\times 10^{-1}$	$9.19\times 10^{-1}$	$5.21\times 10^{-1}$	$6.05\times 10^{-1}$	$1.53\times 10^{-1}$	$1.27\times 10^{-2}$	NA
**Randomized data 3**																	
	**Cow01**	**Cow02**	**Cow03**	**Cow04**	**Cow05**	**Cow06**	**Cow07**	**Cow08**	**Cow09**	**Cow10**	**Cow11**	**Cow12**	**Cow13**	**Cow14**	**Cow15**	**Cow16**	**Cow17**
**Cow01**	NA	$1.84\times 10^{-1}$	$7.19\times 10^{-1}$	$4.06\times 10^{-1}$	$3.88\times 10^{-1}$	$7.69\times 10^{-1}$	$2.22\times 10^{-1}$	$1.91\times 10^{-1}$	$8.04\times 10^{-2}$	$9.47\times 10^{-1}$	$1.96\times 10^{-1}$	$3.07\times 10^{-1}$	$9.93\times 10^{-1}$	$7.30\times 10^{-1}$	$9.80\times 10^{-1}$	$9.47\times 10^{-1}$	$5.38\times 10^{-1}$
**Cow02**	$1.84\times 10^{-1}$	NA	$5.50\times 10^{-2}$	$2.55\times 10^{-2}$	$5.36\times 10^{-1}$	$9.93\times 10^{-2}$	$1.00\times 10^{0}$	$9.70\times 10^{-1}$	$8.31\times 10^{-1}$	$1.13\times 10^{-1}$	$8.98\times 10^{-1}$	$6.22\times 10^{-1}$	$1.41\times 10^{-1}$	$2.89\times 10^{-1}$	$1.77\times 10^{-1}$	$1.66\times 10^{-1}$	$5.15\times 10^{-2}$
**Cow03**	$7.19\times 10^{-1}$	$5.50\times 10^{-2}$	NA	$5.46\times 10^{-1}$	$1.48\times 10^{-1}$	$7.96\times 10^{-1}$	$1.01\times 10^{-1}$	$9.27\times 10^{-2}$	$2.02\times 10^{-2}$	$4.90\times 10^{-1}$	$4.11\times 10^{-2}$	$9.95\times 10^{-2}$	$5.90\times 10^{-1}$	$3.61\times 10^{-1}$	$6.80\times 10^{-1}$	$6.08\times 10^{-1}$	$8.18\times 10^{-1}$
**Cow04**	$4.06\times 10^{-1}$	$2.55\times 10^{-2}$	$5.46\times 10^{-1}$	NA	$6.62\times 10^{-2}$	$5.16\times 10^{-1}$	$4.56\times 10^{-2}$	$3.37\times 10^{-2}$	$7.26\times 10^{-3}$	$2.49\times 10^{-1}$	$1.92\times 10^{-2}$	$4.54\times 10^{-2}$	$3.01\times 10^{-1}$	$2.01\times 10^{-1}$	$3.72\times 10^{-1}$	$3.36\times 10^{-1}$	$8.13\times 10^{-1}$
**Cow05**	$3.88\times 10^{-1}$	$5.36\times 10^{-1}$	$1.48\times 10^{-1}$	$6.62\times 10^{-2}$	NA	$2.96\times 10^{-1}$	$6.21\times 10^{-1}$	$6.50\times 10^{-1}$	$4.09\times 10^{-1}$	$3.57\times 10^{-1}$	$5.60\times 10^{-1}$	$8.80\times 10^{-1}$	$3.70\times 10^{-1}$	$6.47\times 10^{-1}$	$3.88\times 10^{-1}$	$3.97\times 10^{-1}$	$1.45\times 10^{-1}$
**Cow06**	$7.69\times 10^{-1}$	$9.93\times 10^{-2}$	$7.96\times 10^{-1}$	$5.16\times 10^{-1}$	$2.96\times 10^{-1}$	NA	$1.34\times 10^{-1}$	$1.49\times 10^{-1}$	$4.83\times 10^{-2}$	$8.02\times 10^{-1}$	$1.17\times 10^{-1}$	$2.17\times 10^{-1}$	$8.35\times 10^{-1}$	$5.18\times 10^{-1}$	$7.84\times 10^{-1}$	$7.71\times 10^{-1}$	$7.50\times 10^{-1}$
**Cow07**	$2.22\times 10^{-1}$	$1.00\times 10^{0}$	$1.01\times 10^{-1}$	$4.56\times 10^{-2}$	$6.21\times 10^{-1}$	$1.34\times 10^{-1}$	NA	$9.74\times 10^{-1}$	$7.95\times 10^{-1}$	$1.81\times 10^{-1}$	$9.67\times 10^{-1}$	$7.11\times 10^{-1}$	$2.00\times 10^{-1}$	$3.44\times 10^{-1}$	$2.28\times 10^{-1}$	$2.23\times 10^{-1}$	$7.41\times 10^{-2}$
**Cow08**	$1.91\times 10^{-1}$	$9.70\times 10^{-1}$	$9.27\times 10^{-2}$	$3.37\times 10^{-2}$	$6.50\times 10^{-1}$	$1.49\times 10^{-1}$	$9.74\times 10^{-1}$	NA	$7.40\times 10^{-1}$	$2.14\times 10^{-1}$	$9.64\times 10^{-1}$	$7.88\times 10^{-1}$	$2.03\times 10^{-1}$	$3.62\times 10^{-1}$	$2.11\times 10^{-1}$	$2.29\times 10^{-1}$	$6.28\times 10^{-2}$
**Cow09**	$8.04\times 10^{-2}$	$8.31\times 10^{-1}$	$2.02\times 10^{-2}$	$7.26\times 10^{-3}$	$4.09\times 10^{-1}$	$4.83\times 10^{-2}$	$7.95\times 10^{-1}$	$7.40\times 10^{-1}$	NA	$6.09\times 10^{-2}$	$6.80\times 10^{-1}$	$4.91\times 10^{-1}$	$6.57\times 10^{-2}$	$1.78\times 10^{-1}$	$8.76\times 10^{-2}$	$9.29\times 10^{-2}$	$1.89\times 10^{-2}$
**Cow10**	$9.47\times 10^{-1}$	$1.13\times 10^{-1}$	$4.90\times 10^{-1}$	$2.49\times 10^{-1}$	$3.57\times 10^{-1}$	$8.02\times 10^{-1}$	$1.81\times 10^{-1}$	$2.14\times 10^{-1}$	$6.09\times 10^{-2}$	NA	$1.35\times 10^{-1}$	$2.65\times 10^{-1}$	$9.40\times 10^{-1}$	$6.69\times 10^{-1}$	$9.53\times 10^{-1}$	$1.00\times 10^{0}$	$4.74\times 10^{-1}$
**Cow11**	$1.96\times 10^{-1}$	$8.98\times 10^{-1}$	$4.11\times 10^{-2}$	$1.92\times 10^{-2}$	$5.60\times 10^{-1}$	$1.17\times 10^{-1}$	$9.67\times 10^{-1}$	$9.64\times 10^{-1}$	$6.80\times 10^{-1}$	$1.35\times 10^{-1}$	NA	$7.27\times 10^{-1}$	$1.28\times 10^{-1}$	$3.54\times 10^{-1}$	$1.79\times 10^{-1}$	$1.92\times 10^{-1}$	$5.14\times 10^{-2}$
**Cow12**	$3.07\times 10^{-1}$	$6.22\times 10^{-1}$	$9.95\times 10^{-2}$	$4.54\times 10^{-2}$	$8.80\times 10^{-1}$	$2.17\times 10^{-1}$	$7.11\times 10^{-1}$	$7.88\times 10^{-1}$	$4.91\times 10^{-1}$	$2.65\times 10^{-1}$	$7.27\times 10^{-1}$	NA	$2.67\times 10^{-1}$	$5.44\times 10^{-1}$	$3.12\times 10^{-1}$	$3.22\times 10^{-1}$	$1.06\times 10^{-1}$
**Cow13**	$9.93\times 10^{-1}$	$1.41\times 10^{-1}$	$5.90\times 10^{-1}$	$3.01\times 10^{-1}$	$3.70\times 10^{-1}$	$8.35\times 10^{-1}$	$2.00\times 10^{-1}$	$2.03\times 10^{-1}$	$6.57\times 10^{-2}$	$9.40\times 10^{-1}$	$1.28\times 10^{-1}$	$2.67\times 10^{-1}$	NA	$6.56\times 10^{-1}$	$9.93\times 10^{-1}$	$9.70\times 10^{-1}$	$5.21\times 10^{-1}$
**Cow14**	$7.30\times 10^{-1}$	$2.89\times 10^{-1}$	$3.61\times 10^{-1}$	$2.01\times 10^{-1}$	$6.47\times 10^{-1}$	$5.18\times 10^{-1}$	$3.44\times 10^{-1}$	$3.62\times 10^{-1}$	$1.78\times 10^{-1}$	$6.69\times 10^{-1}$	$3.54\times 10^{-1}$	$5.44\times 10^{-1}$	$6.56\times 10^{-1}$	NA	$7.19\times 10^{-1}$	$7.39\times 10^{-1}$	$3.32\times 10^{-1}$
**Cow15**	$9.80\times 10^{-1}$	$1.77\times 10^{-1}$	$6.80\times 10^{-1}$	$3.72\times 10^{-1}$	$3.88\times 10^{-1}$	$7.84\times 10^{-1}$	$2.28\times 10^{-1}$	$2.11\times 10^{-1}$	$8.76\times 10^{-2}$	$9.53\times 10^{-1}$	$1.79\times 10^{-1}$	$3.12\times 10^{-1}$	$9.93\times 10^{-1}$	$7.19\times 10^{-1}$	NA	$9.77\times 10^{-1}$	$5.36\times 10^{-1}$
**Cow16**	$9.47\times 10^{-1}$	$1.66\times 10^{-1}$	$6.08\times 10^{-1}$	$3.36\times 10^{-1}$	$3.97\times 10^{-1}$	$7.71\times 10^{-1}$	$2.23\times 10^{-1}$	$2.29\times 10^{-1}$	$9.29\times 10^{-2}$	$1.00\times 10^{0}$	$1.92\times 10^{-1}$	$3.22\times 10^{-1}$	$9.70\times 10^{-1}$	$7.39\times 10^{-1}$	$9.77\times 10^{-1}$	NA	$5.10\times 10^{-1}$
**Cow17**	$5.38\times 10^{-1}$	$5.15\times 10^{-2}$	$8.18\times 10^{-1}$	$8.13\times 10^{-1}$	$1.45\times 10^{-1}$	$7.50\times 10^{-1}$	$7.41\times 10^{-2}$	$6.28\times 10^{-2}$	$1.89\times 10^{-2}$	$4.74\times 10^{-1}$	$5.14\times 10^{-2}$	$1.06\times 10^{-1}$	$5.21\times 10^{-1}$	$3.32\times 10^{-1}$	$5.36\times 10^{-1}$	$5.10\times 10^{-1}$	NA
**Randomized data 4**																	
	**Cow01**	**Cow02**	**Cow03**	**Cow04**	**Cow05**	**Cow06**	**Cow07**	**Cow08**	**Cow09**	**Cow10**	**Cow11**	**Cow12**	**Cow13**	**Cow14**	**Cow15**	**Cow16**	**Cow17**
**Cow01**	NA	$3.01\times 10^{-1}$	$7.16\times 10^{-1}$	$9.97\times 10^{-1}$	$5.72\times 10^{-1}$	$3.87\times 10^{-1}$	$8.36\times 10^{-1}$	$4.98\times 10^{-1}$	$4.68\times 10^{-1}$	$7.08\times 10^{-1}$	$6.20\times 10^{-1}$	$3.69\times 10^{-1}$	$9.08\times 10^{-1}$	$2.56\times 10^{-1}$	$7.76\times 10^{-1}$	$1.58\times 10^{-1}$	$9.28\times 10^{-1}$
**Cow02**	$3.01\times 10^{-1}$	NA	$1.07\times 10^{-1}$	$2.06\times 10^{-1}$	$1.10\times 10^{-1}$	$4.34\times 10^{-2}$	$1.88\times 10^{-1}$	$6.96\times 10^{-2}$	$5.64\times 10^{-2}$	$1.09\times 10^{-1}$	$4.92\times 10^{-1}$	$3.64\times 10^{-2}$	$2.02\times 10^{-1}$	$1.86\times 10^{-2}$	$1.15\times 10^{-1}$	$5.94\times 10^{-3}$	$2.16\times 10^{-1}$
**Cow03**	$7.16\times 10^{-1}$	$1.07\times 10^{-1}$	NA	$5.71\times 10^{-1}$	$7.52\times 10^{-1}$	$6.35\times 10^{-1}$	$8.61\times 10^{-1}$	$7.47\times 10^{-1}$	$5.93\times 10^{-1}$	$9.76\times 10^{-1}$	$2.96\times 10^{-1}$	$5.04\times 10^{-1}$	$7.92\times 10^{-1}$	$3.02\times 10^{-1}$	$8.65\times 10^{-1}$	$2.79\times 10^{-1}$	$7.79\times 10^{-1}$
**Cow04**	$9.97\times 10^{-1}$	$2.06\times 10^{-1}$	$5.71\times 10^{-1}$	NA	$4.88\times 10^{-1}$	$3.27\times 10^{-1}$	$8.12\times 10^{-1}$	$4.09\times 10^{-1}$	$2.94\times 10^{-1}$	$5.94\times 10^{-1}$	$5.99\times 10^{-1}$	$2.28\times 10^{-1}$	$8.40\times 10^{-1}$	$1.10\times 10^{-1}$	$6.54\times 10^{-1}$	$7.12\times 10^{-2}$	$8.44\times 10^{-1}$
**Cow05**	$5.72\times 10^{-1}$	$1.10\times 10^{-1}$	$7.52\times 10^{-1}$	$4.88\times 10^{-1}$	NA	$8.26\times 10^{-1}$	$7.16\times 10^{-1}$	$9.84\times 10^{-1}$	$9.61\times 10^{-1}$	$7.43\times 10^{-1}$	$2.36\times 10^{-1}$	$8.26\times 10^{-1}$	$6.14\times 10^{-1}$	$6.76\times 10^{-1}$	$6.60\times 10^{-1}$	$5.53\times 10^{-1}$	$5.64\times 10^{-1}$
**Cow06**	$3.87\times 10^{-1}$	$4.34\times 10^{-2}$	$6.35\times 10^{-1}$	$3.27\times 10^{-1}$	$8.26\times 10^{-1}$	NA	$5.08\times 10^{-1}$	$8.87\times 10^{-1}$	$9.93\times 10^{-1}$	$6.31\times 10^{-1}$	$1.54\times 10^{-1}$	$8.90\times 10^{-1}$	$4.52\times 10^{-1}$	$6.36\times 10^{-1}$	$5.44\times 10^{-1}$	$5.34\times 10^{-1}$	$4.70\times 10^{-1}$
**Cow07**	$8.36\times 10^{-1}$	$1.88\times 10^{-1}$	$8.61\times 10^{-1}$	$8.12\times 10^{-1}$	$7.16\times 10^{-1}$	$5.08\times 10^{-1}$	NA	$6.61\times 10^{-1}$	$5.77\times 10^{-1}$	$9.20\times 10^{-1}$	$4.34\times 10^{-1}$	$4.64\times 10^{-1}$	$9.37\times 10^{-1}$	$3.33\times 10^{-1}$	$9.67\times 10^{-1}$	$2.43\times 10^{-1}$	$8.85\times 10^{-1}$
**Cow08**	$4.98\times 10^{-1}$	$6.96\times 10^{-2}$	$7.47\times 10^{-1}$	$4.09\times 10^{-1}$	$9.84\times 10^{-1}$	$8.87\times 10^{-1}$	$6.61\times 10^{-1}$	NA	$9.05\times 10^{-1}$	$7.26\times 10^{-1}$	$1.96\times 10^{-1}$	$7.90\times 10^{-1}$	$5.73\times 10^{-1}$	$5.81\times 10^{-1}$	$6.45\times 10^{-1}$	$5.12\times 10^{-1}$	$5.37\times 10^{-1}$
**Cow09**	$4.68\times 10^{-1}$	$5.64\times 10^{-2}$	$5.93\times 10^{-1}$	$2.94\times 10^{-1}$	$9.61\times 10^{-1}$	$9.93\times 10^{-1}$	$5.77\times 10^{-1}$	$9.05\times 10^{-1}$	NA	$5.86\times 10^{-1}$	$1.36\times 10^{-1}$	$9.07\times 10^{-1}$	$4.85\times 10^{-1}$	$6.79\times 10^{-1}$	$4.85\times 10^{-1}$	$7.01\times 10^{-1}$	$4.45\times 10^{-1}$
**Cow10**	$7.08\times 10^{-1}$	$1.09\times 10^{-1}$	$9.76\times 10^{-1}$	$5.94\times 10^{-1}$	$7.43\times 10^{-1}$	$6.31\times 10^{-1}$	$9.20\times 10^{-1}$	$7.26\times 10^{-1}$	$5.86\times 10^{-1}$	NA	$3.09\times 10^{-1}$	$5.14\times 10^{-1}$	$8.18\times 10^{-1}$	$3.18\times 10^{-1}$	$9.05\times 10^{-1}$	$2.84\times 10^{-1}$	$7.83\times 10^{-1}$
**Cow11**	$6.20\times 10^{-1}$	$4.92\times 10^{-1}$	$2.96\times 10^{-1}$	$5.99\times 10^{-1}$	$2.36\times 10^{-1}$	$1.54\times 10^{-1}$	$4.34\times 10^{-1}$	$1.96\times 10^{-1}$	$1.36\times 10^{-1}$	$3.09\times 10^{-1}$	NA	$1.02\times 10^{-1}$	$4.72\times 10^{-1}$	$4.71\times 10^{-2}$	$3.40\times 10^{-1}$	$2.98\times 10^{-2}$	$5.01\times 10^{-1}$
**Cow12**	$3.69\times 10^{-1}$	$3.64\times 10^{-2}$	$5.04\times 10^{-1}$	$2.28\times 10^{-1}$	$8.26\times 10^{-1}$	$8.90\times 10^{-1}$	$4.64\times 10^{-1}$	$7.90\times 10^{-1}$	$9.07\times 10^{-1}$	$5.14\times 10^{-1}$	$1.02\times 10^{-1}$	NA	$3.87\times 10^{-1}$	$7.37\times 10^{-1}$	$3.99\times 10^{-1}$	$7.35\times 10^{-1}$	$3.76\times 10^{-1}$
**Cow13**	$9.08\times 10^{-1}$	$2.02\times 10^{-1}$	$7.92\times 10^{-1}$	$8.40\times 10^{-1}$	$6.14\times 10^{-1}$	$4.52\times 10^{-1}$	$9.37\times 10^{-1}$	$5.73\times 10^{-1}$	$4.85\times 10^{-1}$	$8.18\times 10^{-1}$	$4.72\times 10^{-1}$	$3.87\times 10^{-1}$	NA	$2.47\times 10^{-1}$	$8.96\times 10^{-1}$	$1.86\times 10^{-1}$	$9.67\times 10^{-1}$
**Cow14**	$2.56\times 10^{-1}$	$1.86\times 10^{-2}$	$3.02\times 10^{-1}$	$1.10\times 10^{-1}$	$6.76\times 10^{-1}$	$6.36\times 10^{-1}$	$3.33\times 10^{-1}$	$5.81\times 10^{-1}$	$6.79\times 10^{-1}$	$3.18\times 10^{-1}$	$4.71\times 10^{-2}$	$7.37\times 10^{-1}$	$2.47\times 10^{-1}$	NA	$2.19\times 10^{-1}$	$9.09\times 10^{-1}$	$2.31\times 10^{-1}$
**Cow15**	$7.76\times 10^{-1}$	$1.15\times 10^{-1}$	$8.65\times 10^{-1}$	$6.54\times 10^{-1}$	$6.60\times 10^{-1}$	$5.44\times 10^{-1}$	$9.67\times 10^{-1}$	$6.45\times 10^{-1}$	$4.85\times 10^{-1}$	$9.05\times 10^{-1}$	$3.40\times 10^{-1}$	$3.99\times 10^{-1}$	$8.96\times 10^{-1}$	$2.19\times 10^{-1}$	NA	$1.95\times 10^{-1}$	$8.63\times 10^{-1}$
**Cow16**	$1.58\times 10^{-1}$	$5.94\times 10^{-3}$	$2.79\times 10^{-1}$	$7.12\times 10^{-2}$	$5.53\times 10^{-1}$	$5.34\times 10^{-1}$	$2.43\times 10^{-1}$	$5.12\times 10^{-1}$	$7.01\times 10^{-1}$	$2.84\times 10^{-1}$	$2.98\times 10^{-2}$	$7.35\times 10^{-1}$	$1.86\times 10^{-1}$	$9.09\times 10^{-1}$	$1.95\times 10^{-1}$	NA	$1.94\times 10^{-1}$
**Cow17**	$9.28\times 10^{-1}$	$2.16\times 10^{-1}$	$7.79\times 10^{-1}$	$8.44\times 10^{-1}$	$5.64\times 10^{-1}$	$4.70\times 10^{-1}$	$8.85\times 10^{-1}$	$5.37\times 10^{-1}$	$4.45\times 10^{-1}$	$7.83\times 10^{-1}$	$5.01\times 10^{-1}$	$3.76\times 10^{-1}$	$9.67\times 10^{-1}$	$2.31\times 10^{-1}$	$8.63\times 10^{-1}$	$1.94\times 10^{-1}$	NA
**Randomized data 5**																	
	**Cow01**	**Cow02**	**Cow03**	**Cow04**	**Cow05**	**Cow06**	**Cow07**	**Cow08**	**Cow09**	**Cow10**	**Cow11**	**Cow12**	**Cow13**	**Cow14**	**Cow15**	**Cow16**	**Cow17**
**Cow01**	NA	$4.41\times 10^{-1}$	$5.72\times 10^{-1}$	$3.41\times 10^{-1}$	$4.74\times 10^{-1}$	$5.34\times 10^{-1}$	$6.92\times 10^{-1}$	$3.34\times 10^{-1}$	$4.09\times 10^{-1}$	$2.66\times 10^{-1}$	$9.37\times 10^{-1}$	$2.49\times 10^{-1}$	$5.94\times 10^{-1}$	$2.67\times 10^{-1}$	$6.41\times 10^{-1}$	$4.43\times 10^{-1}$	$8.21\times 10^{-1}$
**Cow02**	$4.41\times 10^{-1}$	NA	$2.28\times 10^{-1}$	$9.64\times 10^{-1}$	$8.71\times 10^{-1}$	$2.03\times 10^{-1}$	$7.03\times 10^{-1}$	$1.16\times 10^{-1}$	$9.90\times 10^{-1}$	$8.15\times 10^{-2}$	$4.24\times 10^{-1}$	$7.61\times 10^{-1}$	$8.72\times 10^{-1}$	$9.74\times 10^{-2}$	$2.56\times 10^{-1}$	$1.55\times 10^{-1}$	$3.38\times 10^{-1}$
**Cow03**	$5.72\times 10^{-1}$	$2.28\times 10^{-1}$	NA	$1.34\times 10^{-1}$	$2.04\times 10^{-1}$	$9.79\times 10^{-1}$	$3.36\times 10^{-1}$	$7.03\times 10^{-1}$	$1.75\times 10^{-1}$	$5.68\times 10^{-1}$	$7.08\times 10^{-1}$	$1.03\times 10^{-1}$	$3.22\times 10^{-1}$	$5.44\times 10^{-1}$	$9.70\times 10^{-1}$	$8.62\times 10^{-1}$	$7.30\times 10^{-1}$
**Cow04**	$3.41\times 10^{-1}$	$9.64\times 10^{-1}$	$1.34\times 10^{-1}$	NA	$7.92\times 10^{-1}$	$9.00\times 10^{-2}$	$5.77\times 10^{-1}$	$3.64\times 10^{-2}$	$9.70\times 10^{-1}$	$2.44\times 10^{-2}$	$2.83\times 10^{-1}$	$8.27\times 10^{-1}$	$6.51\times 10^{-1}$	$2.85\times 10^{-2}$	$1.19\times 10^{-1}$	$5.99\times 10^{-2}$	$3.01\times 10^{-1}$
**Cow05**	$4.74\times 10^{-1}$	$8.71\times 10^{-1}$	$2.04\times 10^{-1}$	$7.92\times 10^{-1}$	NA	$1.59\times 10^{-1}$	$7.66\times 10^{-1}$	$6.64\times 10^{-2}$	$8.28\times 10^{-1}$	$4.59\times 10^{-2}$	$3.82\times 10^{-1}$	$6.26\times 10^{-1}$	$8.39\times 10^{-1}$	$4.99\times 10^{-2}$	$1.97\times 10^{-1}$	$1.05\times 10^{-1}$	$4.15\times 10^{-1}$
**Cow06**	$5.34\times 10^{-1}$	$2.03\times 10^{-1}$	$9.79\times 10^{-1}$	$9.00\times 10^{-2}$	$1.59\times 10^{-1}$	NA	$2.89\times 10^{-1}$	$6.42\times 10^{-1}$	$1.32\times 10^{-1}$	$4.76\times 10^{-1}$	$7.27\times 10^{-1}$	$8.02\times 10^{-2}$	$3.23\times 10^{-1}$	$4.64\times 10^{-1}$	$9.66\times 10^{-1}$	$8.05\times 10^{-1}$	$6.39\times 10^{-1}$
**Cow07**	$6.92\times 10^{-1}$	$7.03\times 10^{-1}$	$3.36\times 10^{-1}$	$5.77\times 10^{-1}$	$7.66\times 10^{-1}$	$2.89\times 10^{-1}$	NA	$1.51\times 10^{-1}$	$6.34\times 10^{-1}$	$1.11\times 10^{-1}$	$5.76\times 10^{-1}$	$4.58\times 10^{-1}$	$9.49\times 10^{-1}$	$1.10\times 10^{-1}$	$3.50\times 10^{-1}$	$2.22\times 10^{-1}$	$5.76\times 10^{-1}$
**Cow08**	$3.34\times 10^{-1}$	$1.16\times 10^{-1}$	$7.03\times 10^{-1}$	$3.64\times 10^{-2}$	$6.64\times 10^{-2}$	$6.42\times 10^{-1}$	$1.51\times 10^{-1}$	NA	$7.06\times 10^{-2}$	$8.47\times 10^{-1}$	$4.26\times 10^{-1}$	$3.19\times 10^{-2}$	$1.51\times 10^{-1}$	$8.17\times 10^{-1}$	$6.58\times 10^{-1}$	$8.43\times 10^{-1}$	$4.84\times 10^{-1}$
**Cow09**	$4.09\times 10^{-1}$	$9.90\times 10^{-1}$	$1.75\times 10^{-1}$	$9.70\times 10^{-1}$	$8.28\times 10^{-1}$	$1.32\times 10^{-1}$	$6.34\times 10^{-1}$	$7.06\times 10^{-2}$	NA	$4.72\times 10^{-2}$	$3.74\times 10^{-1}$	$7.91\times 10^{-1}$	$7.40\times 10^{-1}$	5.63 × 10^−2^	$1.80\times 10^{-1}$	$1.02\times 10^{-1}$	$3.35\times 10^{-1}$
**Cow10**	$2.66\times 10^{-1}$	$8.15\times 10^{-2}$	$5.68\times 10^{-1}$	$2.44\times 10^{-2}$	$4.59\times 10^{-2}$	$4.76\times 10^{-1}$	$1.11\times 10^{-1}$	$8.47\times 10^{-1}$	$4.72\times 10^{-2}$	NA	$3.29\times 10^{-1}$	$1.91\times 10^{-2}$	$1.04\times 10^{-1}$	$9.97\times 10^{-1}$	$5.09\times 10^{-1}$	$6.92\times 10^{-1}$	$3.80\times 10^{-1}$
**Cow11**	$9.37\times 10^{-1}$	$4.24\times 10^{-1}$	$7.08\times 10^{-1}$	$2.83\times 10^{-1}$	$3.82\times 10^{-1}$	$7.27\times 10^{-1}$	$5.76\times 10^{-1}$	$4.26\times 10^{-1}$	$3.74\times 10^{-1}$	$3.29\times 10^{-1}$	NA	$2.02\times 10^{-1}$	$4.66\times 10^{-1}$	$3.54\times 10^{-1}$	$7.40\times 10^{-1}$	$5.34\times 10^{-1}$	$9.00\times 10^{-1}$
**Cow12**	$2.49\times 10^{-1}$	$7.61\times 10^{-1}$	$1.03\times 10^{-1}$	$8.27\times 10^{-1}$	$6.26\times 10^{-1}$	$8.02\times 10^{-2}$	$4.58\times 10^{-1}$	$3.19\times 10^{-2}$	$7.91\times 10^{-1}$	$1.91\times 10^{-2}$	$2.02\times 10^{-1}$	NA	$5.36\times 10^{-1}$	$2.39\times 10^{-2}$	$9.42\times 10^{-2}$	$4.82\times 10^{-2}$	$2.12\times 10^{-1}$
**Cow13**	$5.94\times 10^{-1}$	$8.72\times 10^{-1}$	$3.22\times 10^{-1}$	$6.51\times 10^{-1}$	$8.39\times 10^{-1}$	$3.23\times 10^{-1}$	$9.49\times 10^{-1}$	$1.51\times 10^{-1}$	$7.40\times 10^{-1}$	$1.04\times 10^{-1}$	$4.66\times 10^{-1}$	$5.36\times 10^{-1}$	NA	$1.07\times 10^{-1}$	$3.27\times 10^{-1}$	$2.12\times 10^{-1}$	$4.85\times 10^{-1}$
**Cow14**	$2.67\times 10^{-1}$	$9.74\times 10^{-2}$	$5.44\times 10^{-1}$	$2.85\times 10^{-2}$	$4.99\times 10^{-2}$	$4.64\times 10^{-1}$	$1.10\times 10^{-1}$	$8.17\times 10^{-1}$	5.63 × 10^−2^	$9.97\times 10^{-1}$	$3.54\times 10^{-1}$	$2.39\times 10^{-2}$	$1.07\times 10^{-1}$	NA	$5.27\times 10^{-1}$	$6.69\times 10^{-1}$	$3.86\times 10^{-1}$
**Cow15**	$6.41\times 10^{-1}$	$2.56\times 10^{-1}$	$9.70\times 10^{-1}$	$1.19\times 10^{-1}$	$1.97\times 10^{-1}$	$9.66\times 10^{-1}$	$3.50\times 10^{-1}$	$6.58\times 10^{-1}$	$1.80\times 10^{-1}$	$5.09\times 10^{-1}$	$7.40\times 10^{-1}$	$9.42\times 10^{-2}$	$3.27\times 10^{-1}$	$5.27\times 10^{-1}$	NA	$8.03\times 10^{-1}$	$7.84\times 10^{-1}$
**Cow16**	$4.43\times 10^{-1}$	$1.55\times 10^{-1}$	$8.62\times 10^{-1}$	$5.99\times 10^{-2}$	$1.05\times 10^{-1}$	$8.05\times 10^{-1}$	$2.22\times 10^{-1}$	$8.43\times 10^{-1}$	$1.02\times 10^{-1}$	$6.92\times 10^{-1}$	$5.34\times 10^{-1}$	$4.82\times 10^{-2}$	$2.12\times 10^{-1}$	$6.69\times 10^{-1}$	$8.03\times 10^{-1}$	NA	$6.02\times 10^{-1}$
**Cow17**	$8.21\times 10^{-1}$	$3.38\times 10^{-1}$	$7.30\times 10^{-1}$	$3.01\times 10^{-1}$	$4.15\times 10^{-1}$	$6.39\times 10^{-1}$	$5.76\times 10^{-1}$	$4.84\times 10^{-1}$	$3.35\times 10^{-1}$	$3.80\times 10^{-1}$	$9.00\times 10^{-1}$	$2.12\times 10^{-1}$	$4.85\times 10^{-1}$	$3.86\times 10^{-1}$	$7.84\times 10^{-1}$	$6.02\times 10^{-1}$	NA
**Randomized data 6**																	
	**Cow01**	**Cow02**	**Cow03**	**Cow04**	**Cow05**	**Cow06**	**Cow07**	**Cow08**	**Cow09**	**Cow10**	**Cow11**	**Cow12**	**Cow13**	**Cow14**	**Cow15**	**Cow16**	**Cow17**
**Cow01**	NA	$6.99\times 10^{-1}$	$7.24\times 10^{-1}$	$1.12\times 10^{-1}$	$7.48\times 10^{-1}$	$7.29\times 10^{-1}$	$2.98\times 10^{-1}$	$2.35\times 10^{-1}$	$9.97\times 10^{-1}$	$5.09\times 10^{-1}$	$1.94\times 10^{-1}$	$6.35\times 10^{-1}$	$3.69\times 10^{-1}$	$9.28\times 10^{-1}$	$6.60\times 10^{-1}$	$1.19\times 10^{-1}$	$2.77\times 10^{-1}$
**Cow02**	$6.99\times 10^{-1}$	NA	$7.23\times 10^{-1}$	$2.63\times 10^{-1}$	$4.18\times 10^{-1}$	$9.97\times 10^{-1}$	$1.73\times 10^{-1}$	$4.22\times 10^{-1}$	$6.17\times 10^{-1}$	$7.85\times 10^{-1}$	$4.31\times 10^{-1}$	$3.63\times 10^{-1}$	$2.50\times 10^{-1}$	$6.83\times 10^{-1}$	$9.97\times 10^{-1}$	$2.60\times 10^{-1}$	$5.40\times 10^{-1}$
**Cow03**	$7.24\times 10^{-1}$	$7.23\times 10^{-1}$	NA	$1.51\times 10^{-1}$	$5.96\times 10^{-1}$	$9.02\times 10^{-1}$	$2.00\times 10^{-1}$	$2.54\times 10^{-1}$	$7.92\times 10^{-1}$	$6.40\times 10^{-1}$	$2.20\times 10^{-1}$	$4.98\times 10^{-1}$	$2.36\times 10^{-1}$	$7.23\times 10^{-1}$	$7.69\times 10^{-1}$	$1.43\times 10^{-1}$	$4.23\times 10^{-1}$
**Cow04**	$1.12\times 10^{-1}$	$2.63\times 10^{-1}$	$1.51\times 10^{-1}$	NA	$6.02\times 10^{-2}$	$2.40\times 10^{-1}$	$1.46\times 10^{-2}$	$7.50\times 10^{-1}$	$1.04\times 10^{-1}$	$3.16\times 10^{-1}$	$7.12\times 10^{-1}$	$4.59\times 10^{-2}$	$1.88\times 10^{-2}$	$1.19\times 10^{-1}$	$2.37\times 10^{-1}$	$8.78\times 10^{-1}$	$5.27\times 10^{-1}$
**Cow05**	$7.48\times 10^{-1}$	$4.18\times 10^{-1}$	$5.96\times 10^{-1}$	$6.02\times 10^{-2}$	NA	$4.97\times 10^{-1}$	$4.84\times 10^{-1}$	$1.18\times 10^{-1}$	$7.75\times 10^{-1}$	$2.85\times 10^{-1}$	$9.30\times 10^{-2}$	$8.96\times 10^{-1}$	$5.91\times 10^{-1}$	$8.18\times 10^{-1}$	$4.17\times 10^{-1}$	$4.72\times 10^{-2}$	$1.69\times 10^{-1}$
**Cow06**	$7.29\times 10^{-1}$	$9.97\times 10^{-1}$	$9.02\times 10^{-1}$	$2.40\times 10^{-1}$	$4.97\times 10^{-1}$	NA	$1.78\times 10^{-1}$	$4.01\times 10^{-1}$	$6.90\times 10^{-1}$	$7.93\times 10^{-1}$	$3.85\times 10^{-1}$	$4.15\times 10^{-1}$	$2.35\times 10^{-1}$	$6.72\times 10^{-1}$	$9.77\times 10^{-1}$	$2.74\times 10^{-1}$	$5.18\times 10^{-1}$
**Cow07**	$2.98\times 10^{-1}$	$1.73\times 10^{-1}$	$2.00\times 10^{-1}$	$1.46\times 10^{-2}$	$4.84\times 10^{-1}$	$1.78\times 10^{-1}$	NA	$2.95\times 10^{-2}$	$3.31\times 10^{-1}$	$1.01\times 10^{-1}$	$2.65\times 10^{-2}$	$5.65\times 10^{-1}$	$8.37\times 10^{-1}$	$3.73\times 10^{-1}$	$1.54\times 10^{-1}$	$1.53\times 10^{-2}$	$4.39\times 10^{-2}$
**Cow08**	$2.35\times 10^{-1}$	$4.22\times 10^{-1}$	$2.54\times 10^{-1}$	$7.50\times 10^{-1}$	$1.18\times 10^{-1}$	$4.01\times 10^{-1}$	$2.95\times 10^{-2}$	NA	$1.85\times 10^{-1}$	$5.57\times 10^{-1}$	$9.97\times 10^{-1}$	$9.20\times 10^{-2}$	$4.23\times 10^{-2}$	$2.15\times 10^{-1}$	$4.09\times 10^{-1}$	$7.61\times 10^{-1}$	$8.05\times 10^{-1}$
**Cow09**	$9.97\times 10^{-1}$	$6.17\times 10^{-1}$	$7.92\times 10^{-1}$	$1.04\times 10^{-1}$	$7.75\times 10^{-1}$	$6.90\times 10^{-1}$	$3.31\times 10^{-1}$	$1.85\times 10^{-1}$	NA	$5.01\times 10^{-1}$	$1.78\times 10^{-1}$	$6.77\times 10^{-1}$	$4.02\times 10^{-1}$	$9.63\times 10^{-1}$	$6.24\times 10^{-1}$	$1.14\times 10^{-1}$	$2.88\times 10^{-1}$
**Cow10**	$5.09\times 10^{-1}$	$7.85\times 10^{-1}$	$6.40\times 10^{-1}$	$3.16\times 10^{-1}$	$2.85\times 10^{-1}$	$7.93\times 10^{-1}$	$1.01\times 10^{-1}$	$5.57\times 10^{-1}$	$5.01\times 10^{-1}$	NA	$5.67\times 10^{-1}$	$2.30\times 10^{-1}$	$1.29\times 10^{-1}$	$5.18\times 10^{-1}$	$7.92\times 10^{-1}$	$3.75\times 10^{-1}$	$6.68\times 10^{-1}$
**Cow11**	$1.94\times 10^{-1}$	$4.31\times 10^{-1}$	$2.20\times 10^{-1}$	$7.12\times 10^{-1}$	$9.30\times 10^{-2}$	$3.85\times 10^{-1}$	$2.65\times 10^{-2}$	$9.97\times 10^{-1}$	$1.78\times 10^{-1}$	$5.67\times 10^{-1}$	NA	$7.22\times 10^{-2}$	$3.54\times 10^{-2}$	$2.12\times 10^{-1}$	$3.99\times 10^{-1}$	$7.20\times 10^{-1}$	$8.10\times 10^{-1}$
**Cow12**	$6.35\times 10^{-1}$	$3.63\times 10^{-1}$	$4.98\times 10^{-1}$	$4.59\times 10^{-2}$	$8.96\times 10^{-1}$	$4.15\times 10^{-1}$	$5.65\times 10^{-1}$	$9.20\times 10^{-2}$	$6.77\times 10^{-1}$	$2.30\times 10^{-1}$	$7.22\times 10^{-2}$	NA	$7.00\times 10^{-1}$	$7.20\times 10^{-1}$	$3.49\times 10^{-1}$	$3.63\times 10^{-2}$	$1.28\times 10^{-1}$
**Cow13**	$3.69\times 10^{-1}$	$2.50\times 10^{-1}$	$2.36\times 10^{-1}$	$1.88\times 10^{-2}$	$5.91\times 10^{-1}$	$2.35\times 10^{-1}$	$8.37\times 10^{-1}$	$4.23\times 10^{-2}$	$4.02\times 10^{-1}$	$1.29\times 10^{-1}$	$3.54\times 10^{-2}$	$7.00\times 10^{-1}$	NA	$4.67\times 10^{-1}$	$2.10\times 10^{-1}$	$2.02\times 10^{-2}$	5.39 × 10^−2^
**Cow14**	$9.28\times 10^{-1}$	$6.83\times 10^{-1}$	$7.23\times 10^{-1}$	$1.19\times 10^{-1}$	$8.18\times 10^{-1}$	$6.72\times 10^{-1}$	$3.73\times 10^{-1}$	$2.15\times 10^{-1}$	$9.63\times 10^{-1}$	$5.18\times 10^{-1}$	$2.12\times 10^{-1}$	$7.20\times 10^{-1}$	$4.67\times 10^{-1}$	NA	$6.54\times 10^{-1}$	$1.53\times 10^{-1}$	$2.88\times 10^{-1}$
**Cow15**	$6.60\times 10^{-1}$	$9.97\times 10^{-1}$	$7.69\times 10^{-1}$	$2.37\times 10^{-1}$	$4.17\times 10^{-1}$	$9.77\times 10^{-1}$	$1.54\times 10^{-1}$	$4.09\times 10^{-1}$	$6.24\times 10^{-1}$	$7.92\times 10^{-1}$	$3.99\times 10^{-1}$	$3.49\times 10^{-1}$	$2.10\times 10^{-1}$	$6.54\times 10^{-1}$	NA	$2.48\times 10^{-1}$	$5.30\times 10^{-1}$
**Cow16**	$1.19\times 10^{-1}$	$2.60\times 10^{-1}$	$1.43\times 10^{-1}$	$8.78\times 10^{-1}$	$4.72\times 10^{-2}$	$2.74\times 10^{-1}$	$1.53\times 10^{-2}$	$7.61\times 10^{-1}$	$1.14\times 10^{-1}$	$3.75\times 10^{-1}$	$7.20\times 10^{-1}$	$3.63\times 10^{-2}$	$2.02\times 10^{-2}$	$1.53\times 10^{-1}$	$2.48\times 10^{-1}$	$6.34\times 10^{-1}$	
**Cow17**	$2.77\times 10^{-1}$	$5.40\times 10^{-1}$	$4.23\times 10^{-1}$	$5.27\times 10^{-1}$	$1.69\times 10^{-1}$	$5.18\times 10^{-1}$	$4.39\times 10^{-2}$	$8.05\times 10^{-1}$	$2.88\times 10^{-1}$	$6.68\times 10^{-1}$	$8.10\times 10^{-1}$	$1.28\times 10^{-1}$	5.39 × 10^−2^	$2.88\times 10^{-1}$	$5.30\times 10^{-1}$	$6.34\times 10^{-1}$	NA
**Randomized data 7**																	
	**Cow01**	**Cow02**	**Cow03**	**Cow04**	**Cow05**	**Cow06**	**Cow07**	**Cow08**	**Cow09**	**Cow10**	**Cow11**	**Cow12**	**Cow13**	**Cow14**	**Cow15**	**Cow16**	**Cow17**
**Cow01**	NA	$7.94\times 10^{-1}$	$5.17\times 10^{-1}$	$7.63\times 10^{-2}$	$9.93\times 10^{-1}$	$6.80\times 10^{-1}$	$4.71\times 10^{-1}$	$8.14\times 10^{-1}$	$3.34\times 10^{-1}$	$8.51\times 10^{-1}$	$6.43\times 10^{-1}$	$9.41\times 10^{-1}$	$2.34\times 10^{-1}$	$4.20\times 10^{-1}$	$2.15\times 10^{-1}$	$2.28\times 10^{-1}$	$9.11\times 10^{-2}$
**Cow02**	$7.94\times 10^{-1}$	NA	$4.22\times 10^{-1}$	$1.42\times 10^{-1}$	$8.91\times 10^{-1}$	$7.69\times 10^{-1}$	$6.15\times 10^{-1}$	$9.97\times 10^{-1}$	$2.97\times 10^{-1}$	$7.72\times 10^{-1}$	$5.63\times 10^{-1}$	$8.57\times 10^{-1}$	$1.89\times 10^{-1}$	$3.15\times 10^{-1}$	$1.83\times 10^{-1}$	$1.91\times 10^{-1}$	$7.39\times 10^{-2}$
**Cow03**	$5.17\times 10^{-1}$	$4.22\times 10^{-1}$	NA	$4.00\times 10^{-2}$	$5.17\times 10^{-1}$	$3.22\times 10^{-1}$	$1.89\times 10^{-1}$	$3.98\times 10^{-1}$	$8.92\times 10^{-1}$	$6.93\times 10^{-1}$	$7.23\times 10^{-1}$	$7.34\times 10^{-1}$	$7.83\times 10^{-1}$	$9.41\times 10^{-1}$	$7.57\times 10^{-1}$	$7.07\times 10^{-1}$	$4.03\times 10^{-1}$
**Cow04**	$7.63\times 10^{-2}$	$1.42\times 10^{-1}$	$4.00\times 10^{-2}$	NA	$1.39\times 10^{-1}$	$3.49\times 10^{-1}$	$3.28\times 10^{-1}$	$1.71\times 10^{-1}$	$1.82\times 10^{-2}$	$1.33\times 10^{-1}$	$4.70\times 10^{-2}$	$2.32\times 10^{-1}$	$5.48\times 10^{-3}$	$1.41\times 10^{-2}$	$6.44\times 10^{-3}$	$1.09\times 10^{-2}$	$1.91\times 10^{-3}$
**Cow05**	$9.93\times 10^{-1}$	$8.91\times 10^{-1}$	$5.17\times 10^{-1}$	$1.39\times 10^{-1}$	NA	$6.88\times 10^{-1}$	$4.97\times 10^{-1}$	$8.39\times 10^{-1}$	$4.12\times 10^{-1}$	$8.16\times 10^{-1}$	$7.61\times 10^{-1}$	$8.37\times 10^{-1}$	$3.36\times 10^{-1}$	$4.44\times 10^{-1}$	$3.13\times 10^{-1}$	$2.91\times 10^{-1}$	$1.15\times 10^{-1}$
**Cow06**	$6.80\times 10^{-1}$	$7.69\times 10^{-1}$	$3.22\times 10^{-1}$	$3.49\times 10^{-1}$	$6.88\times 10^{-1}$	NA	$8.25\times 10^{-1}$	$8.59\times 10^{-1}$	$2.34\times 10^{-1}$	$5.54\times 10^{-1}$	$4.76\times 10^{-1}$	$6.02\times 10^{-1}$	$1.70\times 10^{-1}$	$2.55\times 10^{-1}$	$1.80\times 10^{-1}$	$1.79\times 10^{-1}$	$5.78\times 10^{-2}$
**Cow07**	$4.71\times 10^{-1}$	$6.15\times 10^{-1}$	$1.89\times 10^{-1}$	$3.28\times 10^{-1}$	$4.97\times 10^{-1}$	$8.25\times 10^{-1}$	NA	$6.48\times 10^{-1}$	$1.08\times 10^{-1}$	$3.82\times 10^{-1}$	$2.69\times 10^{-1}$	$4.77\times 10^{-1}$	$7.06\times 10^{-2}$	$1.30\times 10^{-1}$	$6.18\times 10^{-2}$	$6.76\times 10^{-2}$	$1.74\times 10^{-2}$
**Cow08**	$8.14\times 10^{-1}$	$9.97\times 10^{-1}$	$3.98\times 10^{-1}$	$1.71\times 10^{-1}$	$8.39\times 10^{-1}$	$8.59\times 10^{-1}$	$6.48\times 10^{-1}$	NA	$2.69\times 10^{-1}$	$6.79\times 10^{-1}$	$5.43\times 10^{-1}$	$7.30\times 10^{-1}$	$1.96\times 10^{-1}$	$3.19\times 10^{-1}$	$1.77\times 10^{-1}$	$1.80\times 10^{-1}$	$6.83\times 10^{-2}$
**Cow09**	$3.34\times 10^{-1}$	$2.97\times 10^{-1}$	$8.92\times 10^{-1}$	$1.82\times 10^{-2}$	$4.12\times 10^{-1}$	$2.34\times 10^{-1}$	$1.08\times 10^{-1}$	$2.69\times 10^{-1}$	NA	$6.03\times 10^{-1}$	$5.65\times 10^{-1}$	$6.48\times 10^{-1}$	$9.50\times 10^{-1}$	$8.87\times 10^{-1}$	$9.33\times 10^{-1}$	$8.22\times 10^{-1}$	$4.54\times 10^{-1}$
**Cow10**	$8.51\times 10^{-1}$	$7.72\times 10^{-1}$	$6.93\times 10^{-1}$	$1.33\times 10^{-1}$	$8.16\times 10^{-1}$	$5.54\times 10^{-1}$	$3.82\times 10^{-1}$	$6.79\times 10^{-1}$	$6.03\times 10^{-1}$	NA	$9.54\times 10^{-1}$	$9.93\times 10^{-1}$	$4.85\times 10^{-1}$	$6.20\times 10^{-1}$	$5.04\times 10^{-1}$	$4.81\times 10^{-1}$	$2.15\times 10^{-1}$
**Cow11**	$6.43\times 10^{-1}$	$5.63\times 10^{-1}$	$7.23\times 10^{-1}$	$4.70\times 10^{-2}$	$7.61\times 10^{-1}$	$4.76\times 10^{-1}$	$2.69\times 10^{-1}$	$5.43\times 10^{-1}$	$5.65\times 10^{-1}$	$9.54\times 10^{-1}$	NA	$9.31\times 10^{-1}$	$4.93\times 10^{-1}$	$7.03\times 10^{-1}$	$4.94\times 10^{-1}$	$4.26\times 10^{-1}$	$1.87\times 10^{-1}$
**Cow12**	$9.41\times 10^{-1}$	$8.57\times 10^{-1}$	$7.34\times 10^{-1}$	$2.32\times 10^{-1}$	$8.37\times 10^{-1}$	$6.02\times 10^{-1}$	$4.77\times 10^{-1}$	$7.30\times 10^{-1}$	$6.48\times 10^{-1}$	$9.93\times 10^{-1}$	$9.31\times 10^{-1}$	NA	$4.88\times 10^{-1}$	$6.06\times 10^{-1}$	$5.11\times 10^{-1}$	$5.62\times 10^{-1}$	$2.74\times 10^{-1}$
**Cow13**	$2.34\times 10^{-1}$	$1.89\times 10^{-1}$	$7.83\times 10^{-1}$	$5.48\times 10^{-3}$	$3.36\times 10^{-1}$	$1.70\times 10^{-1}$	$7.06\times 10^{-2}$	$1.96\times 10^{-1}$	$9.50\times 10^{-1}$	$4.85\times 10^{-1}$	$4.93\times 10^{-1}$	$4.88\times 10^{-1}$	NA	$7.77\times 10^{-1}$	$9.87\times 10^{-1}$	$8.39\times 10^{-1}$	$5.00\times 10^{-1}$
**Cow14**	$4.20\times 10^{-1}$	$3.15\times 10^{-1}$	$9.41\times 10^{-1}$	$1.41\times 10^{-2}$	$4.44\times 10^{-1}$	$2.55\times 10^{-1}$	$1.30\times 10^{-1}$	$3.19\times 10^{-1}$	$8.87\times 10^{-1}$	$6.20\times 10^{-1}$	$7.03\times 10^{-1}$	$6.06\times 10^{-1}$	$7.77\times 10^{-1}$	NA	$7.19\times 10^{-1}$	$6.79\times 10^{-1}$	$3.78\times 10^{-1}$
**Cow15**	$2.15\times 10^{-1}$	$1.83\times 10^{-1}$	$7.57\times 10^{-1}$	$6.44\times 10^{-3}$	$3.13\times 10^{-1}$	$1.80\times 10^{-1}$	$6.18\times 10^{-2}$	$1.77\times 10^{-1}$	$9.33\times 10^{-1}$	$5.04\times 10^{-1}$	$4.94\times 10^{-1}$	$5.11\times 10^{-1}$	$9.87\times 10^{-1}$	$7.19\times 10^{-1}$	NA	$9.05\times 10^{-1}$	$5.20\times 10^{-1}$
**Cow16**	$2.28\times 10^{-1}$	$1.91\times 10^{-1}$	$7.07\times 10^{-1}$	$1.09\times 10^{-2}$	$2.91\times 10^{-1}$	$1.79\times 10^{-1}$	$6.76\times 10^{-2}$	$1.80\times 10^{-1}$	$8.22\times 10^{-1}$	$4.81\times 10^{-1}$	$4.26\times 10^{-1}$	$5.62\times 10^{-1}$	$8.39\times 10^{-1}$	$6.79\times 10^{-1}$	$9.05\times 10^{-1}$	NA	$6.42\times 10^{-1}$
**Cow17**	$9.11\times 10^{-2}$	$7.39\times 10^{-2}$	$4.03\times 10^{-1}$	$1.91\times 10^{-3}$	$1.15\times 10^{-1}$	$5.78\times 10^{-2}$	$1.74\times 10^{-2}$	$6.83\times 10^{-2}$	$4.54\times 10^{-1}$	$2.15\times 10^{-1}$	$1.87\times 10^{-1}$	$2.74\times 10^{-1}$	$5.00\times 10^{-1}$	$3.78\times 10^{-1}$	$5.20\times 10^{-1}$	$6.42\times 10^{-1}$	NA
**Randomized data 8**																	
	**Cow01**	**Cow02**	**Cow03**	**Cow04**	**Cow05**	**Cow06**	**Cow07**	**Cow08**	**Cow09**	**Cow10**	**Cow11**	**Cow12**	**Cow13**	**Cow14**	**Cow15**	**Cow16**	**Cow17**
**Cow01**	NA	$4.44\times 10^{-1}$	$5.85\times 10^{-1}$	$2.89\times 10^{-1}$	$9.04\times 10^{-1}$	$1.96\times 10^{-1}$	$4.32\times 10^{-1}$	$4.11\times 10^{-1}$	$1.69\times 10^{-1}$	$9.63\times 10^{-1}$	$3.37\times 10^{-1}$	$9.87\times 10^{-1}$	$3.16\times 10^{-2}$	$5.41\times 10^{-1}$	$1.02\times 10^{-1}$	$4.07\times 10^{-1}$	$1.03\times 10^{-1}$
**Cow02**	$4.44\times 10^{-1}$	NA	$8.44\times 10^{-1}$	$9.32\times 10^{-1}$	$5.31\times 10^{-1}$	$5.39\times 10^{-1}$	$9.37\times 10^{-1}$	$9.57\times 10^{-1}$	$5.24\times 10^{-1}$	$3.99\times 10^{-1}$	$7.30\times 10^{-1}$	$3.79\times 10^{-1}$	$1.75\times 10^{-1}$	$9.08\times 10^{-1}$	$4.35\times 10^{-1}$	$8.09\times 10^{-1}$	$3.26\times 10^{-1}$
**Cow03**	$5.85\times 10^{-1}$	$8.44\times 10^{-1}$	NA	$6.62\times 10^{-1}$	$6.51\times 10^{-1}$	$4.10\times 10^{-1}$	$7.67\times 10^{-1}$	$7.96\times 10^{-1}$	$3.84\times 10^{-1}$	$5.63\times 10^{-1}$	$5.82\times 10^{-1}$	$5.11\times 10^{-1}$	$8.98\times 10^{-2}$	$9.67\times 10^{-1}$	$3.02\times 10^{-1}$	$6.78\times 10^{-1}$	$2.39\times 10^{-1}$
**Cow04**	$2.89\times 10^{-1}$	$9.32\times 10^{-1}$	$6.62\times 10^{-1}$	NA	$3.76\times 10^{-1}$	$6.47\times 10^{-1}$	$9.32\times 10^{-1}$	$9.39\times 10^{-1}$	$5.74\times 10^{-1}$	$2.54\times 10^{-1}$	$7.80\times 10^{-1}$	$2.46\times 10^{-1}$	$1.59\times 10^{-1}$	$7.76\times 10^{-1}$	$4.85\times 10^{-1}$	$9.43\times 10^{-1}$	$3.73\times 10^{-1}$
**Cow05**	$9.04\times 10^{-1}$	$5.31\times 10^{-1}$	$6.51\times 10^{-1}$	$3.76\times 10^{-1}$	NA	$2.25\times 10^{-1}$	$4.72\times 10^{-1}$	$5.20\times 10^{-1}$	$2.06\times 10^{-1}$	$9.04\times 10^{-1}$	$3.60\times 10^{-1}$	$8.62\times 10^{-1}$	$4.12\times 10^{-2}$	$6.39\times 10^{-1}$	$1.40\times 10^{-1}$	$4.41\times 10^{-1}$	$1.25\times 10^{-1}$
**Cow06**	$1.96\times 10^{-1}$	$5.39\times 10^{-1}$	$4.10\times 10^{-1}$	$6.47\times 10^{-1}$	$2.25\times 10^{-1}$	NA	$6.08\times 10^{-1}$	$5.72\times 10^{-1}$	$9.90\times 10^{-1}$	$1.49\times 10^{-1}$	$8.45\times 10^{-1}$	$1.47\times 10^{-1}$	$4.23\times 10^{-1}$	$4.39\times 10^{-1}$	$9.05\times 10^{-1}$	$7.10\times 10^{-1}$	$7.84\times 10^{-1}$
**Cow07**	$4.32\times 10^{-1}$	$9.37\times 10^{-1}$	$7.67\times 10^{-1}$	$9.32\times 10^{-1}$	$4.72\times 10^{-1}$	$6.08\times 10^{-1}$	NA	$9.83\times 10^{-1}$	$5.68\times 10^{-1}$	$3.63\times 10^{-1}$	$7.63\times 10^{-1}$	$3.48\times 10^{-1}$	$1.71\times 10^{-1}$	$8.14\times 10^{-1}$	$5.05\times 10^{-1}$	$8.81\times 10^{-1}$	$4.00\times 10^{-1}$
**Cow08**	$4.11\times 10^{-1}$	$9.57\times 10^{-1}$	$7.96\times 10^{-1}$	$9.39\times 10^{-1}$	$5.20\times 10^{-1}$	$5.72\times 10^{-1}$	$9.83\times 10^{-1}$	NA	$5.90\times 10^{-1}$	$4.01\times 10^{-1}$	$8.22\times 10^{-1}$	$3.82\times 10^{-1}$	$2.01\times 10^{-1}$	$8.56\times 10^{-1}$	$4.28\times 10^{-1}$	$8.95\times 10^{-1}$	$3.79\times 10^{-1}$
**Cow09**	$1.69\times 10^{-1}$	$5.24\times 10^{-1}$	$3.84\times 10^{-1}$	$5.74\times 10^{-1}$	$2.06\times 10^{-1}$	$9.90\times 10^{-1}$	$5.68\times 10^{-1}$	$5.90\times 10^{-1}$	NA	$1.32\times 10^{-1}$	$8.03\times 10^{-1}$	$1.27\times 10^{-1}$	$4.47\times 10^{-1}$	$4.51\times 10^{-1}$	$9.04\times 10^{-1}$	$7.10\times 10^{-1}$	$7.46\times 10^{-1}$
**Cow10**	$9.63\times 10^{-1}$	$3.99\times 10^{-1}$	$5.63\times 10^{-1}$	$2.54\times 10^{-1}$	$9.04\times 10^{-1}$	$1.49\times 10^{-1}$	$3.63\times 10^{-1}$	$4.01\times 10^{-1}$	$1.32\times 10^{-1}$	NA	$2.51\times 10^{-1}$	$9.50\times 10^{-1}$	$1.62\times 10^{-2}$	$5.21\times 10^{-1}$	$8.13\times 10^{-2}$	$3.32\times 10^{-1}$	$7.14\times 10^{-2}$
**Cow11**	$3.37\times 10^{-1}$	$7.30\times 10^{-1}$	$5.82\times 10^{-1}$	$7.80\times 10^{-1}$	$3.60\times 10^{-1}$	$8.45\times 10^{-1}$	$7.63\times 10^{-1}$	$8.22\times 10^{-1}$	$8.03\times 10^{-1}$	$2.51\times 10^{-1}$	NA	$2.60\times 10^{-1}$	$3.40\times 10^{-1}$	$6.66\times 10^{-1}$	$7.49\times 10^{-1}$	$9.17\times 10^{-1}$	$6.12\times 10^{-1}$
**Cow12**	$9.87\times 10^{-1}$	$3.79\times 10^{-1}$	$5.11\times 10^{-1}$	$2.46\times 10^{-1}$	$8.62\times 10^{-1}$	$1.47\times 10^{-1}$	$3.48\times 10^{-1}$	$3.82\times 10^{-1}$	$1.27\times 10^{-1}$	$9.50\times 10^{-1}$	$2.60\times 10^{-1}$	NA	$1.90\times 10^{-2}$	$4.88\times 10^{-1}$	$7.36\times 10^{-2}$	$3.33\times 10^{-1}$	$7.12\times 10^{-2}$
**Cow13**	$3.16\times 10^{-2}$	$1.75\times 10^{-1}$	$8.98\times 10^{-2}$	$1.59\times 10^{-1}$	$4.12\times 10^{-2}$	$4.23\times 10^{-1}$	$1.71\times 10^{-1}$	$2.01\times 10^{-1}$	$4.47\times 10^{-1}$	$1.62\times 10^{-2}$	$3.40\times 10^{-1}$	$1.90\times 10^{-2}$	NA	$1.31\times 10^{-1}$	$5.15\times 10^{-1}$	$2.58\times 10^{-1}$	$6.38\times 10^{-1}$
**Cow14**	$5.41\times 10^{-1}$	$9.08\times 10^{-1}$	$9.67\times 10^{-1}$	$7.76\times 10^{-1}$	$6.39\times 10^{-1}$	$4.39\times 10^{-1}$	$8.14\times 10^{-1}$	$8.56\times 10^{-1}$	$4.51\times 10^{-1}$	$5.21\times 10^{-1}$	$6.66\times 10^{-1}$	$4.88\times 10^{-1}$	$1.31\times 10^{-1}$	NA	$3.23\times 10^{-1}$	$7.32\times 10^{-1}$	$2.75\times 10^{-1}$
**Cow15**	$1.02\times 10^{-1}$	$4.35\times 10^{-1}$	$3.02\times 10^{-1}$	$4.85\times 10^{-1}$	$1.40\times 10^{-1}$	$9.05\times 10^{-1}$	$5.05\times 10^{-1}$	$4.28\times 10^{-1}$	$9.04\times 10^{-1}$	$8.13\times 10^{-2}$	$7.49\times 10^{-1}$	$7.36\times 10^{-2}$	$5.15\times 10^{-1}$	$3.23\times 10^{-1}$	NA	$6.14\times 10^{-1}$	$8.16\times 10^{-1}$
**Cow16**	$4.07\times 10^{-1}$	$8.09\times 10^{-1}$	$6.78\times 10^{-1}$	$9.43\times 10^{-1}$	$4.41\times 10^{-1}$	$7.10\times 10^{-1}$	$8.81\times 10^{-1}$	$8.95\times 10^{-1}$	$7.10\times 10^{-1}$	$3.32\times 10^{-1}$	$9.17\times 10^{-1}$	$3.33\times 10^{-1}$	$2.58\times 10^{-1}$	$7.32\times 10^{-1}$	$6.14\times 10^{-1}$	NA	$5.31\times 10^{-1}$
**Cow17**	$1.03\times 10^{-1}$	$3.26\times 10^{-1}$	$2.39\times 10^{-1}$	$3.73\times 10^{-1}$	$1.25\times 10^{-1}$	$7.84\times 10^{-1}$	$4.00\times 10^{-1}$	$3.79\times 10^{-1}$	$7.46\times 10^{-1}$	$7.14\times 10^{-2}$	$6.12\times 10^{-1}$	$7.12\times 10^{-2}$	$6.38\times 10^{-1}$	$2.75\times 10^{-1}$	$8.16\times 10^{-1}$	$5.31\times 10^{-1}$	NA
**Randomized data 9**																	
	**Cow01**	**Cow02**	**Cow03**	**Cow04**	**Cow05**	**Cow06**	**Cow07**	**Cow08**	**Cow09**	**Cow10**	**Cow11**	**Cow12**	**Cow13**	**Cow14**	**Cow15**	**Cow16**	**Cow17**
**Cow01**	NA	$5.50\times 10^{-1}$	$4.28\times 10^{-1}$	$1.12\times 10^{-1}$	$5.05\times 10^{-1}$	$8.43\times 10^{-1}$	$8.91\times 10^{-1}$	$8.17\times 10^{-1}$	$6.26\times 10^{-1}$	$5.48\times 10^{-1}$	$9.90\times 10^{-1}$	$4.12\times 10^{-1}$	$4.88\times 10^{-1}$	$8.69\times 10^{-1}$	$6.21\times 10^{-1}$	$2.98\times 10^{-1}$	$6.39\times 10^{-1}$
**Cow02**	$5.50\times 10^{-1}$	NA	$1.00\times 10^{-1}$	$2.45\times 10^{-1}$	$1.34\times 10^{-1}$	$3.86\times 10^{-1}$	$5.96\times 10^{-1}$	$3.38\times 10^{-1}$	$8.85\times 10^{-1}$	$1.80\times 10^{-1}$	$4.91\times 10^{-1}$	$7.20\times 10^{-1}$	$8.92\times 10^{-1}$	$4.35\times 10^{-1}$	$2.23\times 10^{-1}$	$6.15\times 10^{-1}$	$2.08\times 10^{-1}$
**Cow03**	$4.28\times 10^{-1}$	$1.00\times 10^{-1}$	NA	$5.87\times 10^{-3}$	$7.66\times 10^{-1}$	$5.69\times 10^{-1}$	$3.22\times 10^{-1}$	$5.78\times 10^{-1}$	$9.10\times 10^{-2}$	$7.16\times 10^{-1}$	$3.54\times 10^{-1}$	$7.34\times 10^{-2}$	$6.77\times 10^{-2}$	$5.18\times 10^{-1}$	$7.39\times 10^{-1}$	$3.07\times 10^{-2}$	$7.07\times 10^{-1}$
**Cow04**	$1.12\times 10^{-1}$	$2.45\times 10^{-1}$	$5.87\times 10^{-3}$	NA	$7.07\times 10^{-3}$	$4.38\times 10^{-2}$	$9.93\times 10^{-2}$	$3.57\times 10^{-2}$	$1.02\times 10^{-1}$	$8.13\times 10^{-3}$	$6.56\times 10^{-2}$	$5.55\times 10^{-1}$	$2.73\times 10^{-1}$	$5.07\times 10^{-2}$	$2.36\times 10^{-2}$	$4.72\times 10^{-1}$	$2.86\times 10^{-2}$
**Cow05**	$5.05\times 10^{-1}$	$1.34\times 10^{-1}$	$7.66\times 10^{-1}$	$7.07\times 10^{-3}$	NA	$6.77\times 10^{-1}$	$4.20\times 10^{-1}$	$7.16\times 10^{-1}$	$1.09\times 10^{-1}$	$8.59\times 10^{-1}$	$4.76\times 10^{-1}$	$9.61\times 10^{-2}$	$8.39\times 10^{-2}$	$5.84\times 10^{-1}$	$9.30\times 10^{-1}$	$4.03\times 10^{-2}$	$9.77\times 10^{-1}$
**Cow06**	$8.43\times 10^{-1}$	$3.86\times 10^{-1}$	$5.69\times 10^{-1}$	$4.38\times 10^{-2}$	$6.77\times 10^{-1}$	NA	$7.19\times 10^{-1}$	$9.74\times 10^{-1}$	$4.28\times 10^{-1}$	$7.45\times 10^{-1}$	$7.82\times 10^{-1}$	$2.56\times 10^{-1}$	$3.08\times 10^{-1}$	$9.24\times 10^{-1}$	$8.10\times 10^{-1}$	$1.64\times 10^{-1}$	$7.57\times 10^{-1}$
**Cow07**	$8.91\times 10^{-1}$	$5.96\times 10^{-1}$	$3.22\times 10^{-1}$	$9.93\times 10^{-2}$	$4.20\times 10^{-1}$	$7.19\times 10^{-1}$	NA	$6.87\times 10^{-1}$	$6.39\times 10^{-1}$	$4.75\times 10^{-1}$	$9.17\times 10^{-1}$	$4.23\times 10^{-1}$	$5.04\times 10^{-1}$	$7.78\times 10^{-1}$	$5.17\times 10^{-1}$	$2.99\times 10^{-1}$	$5.21\times 10^{-1}$
**Cow08**	$8.17\times 10^{-1}$	$3.38\times 10^{-1}$	$5.78\times 10^{-1}$	$3.57\times 10^{-2}$	$7.16\times 10^{-1}$	$9.74\times 10^{-1}$	$6.87\times 10^{-1}$	NA	$3.75\times 10^{-1}$	$7.57\times 10^{-1}$	$7.49\times 10^{-1}$	$2.19\times 10^{-1}$	$2.81\times 10^{-1}$	$9.41\times 10^{-1}$	$8.06\times 10^{-1}$	$1.38\times 10^{-1}$	$8.36\times 10^{-1}$
**Cow09**	$6.26\times 10^{-1}$	$8.85\times 10^{-1}$	$9.10\times 10^{-2}$	$1.02\times 10^{-1}$	$1.09\times 10^{-1}$	$4.28\times 10^{-1}$	$6.39\times 10^{-1}$	$3.75\times 10^{-1}$	NA	$1.82\times 10^{-1}$	$5.43\times 10^{-1}$	$5.21\times 10^{-1}$	$7.77\times 10^{-1}$	$5.32\times 10^{-1}$	$2.19\times 10^{-1}$	$4.25\times 10^{-1}$	$1.33\times 10^{-1}$
**Cow10**	$5.48\times 10^{-1}$	$1.80\times 10^{-1}$	$7.16\times 10^{-1}$	$8.13\times 10^{-3}$	$8.59\times 10^{-1}$	$7.45\times 10^{-1}$	$4.75\times 10^{-1}$	$7.57\times 10^{-1}$	$1.82\times 10^{-1}$	NA	$5.35\times 10^{-1}$	$1.02\times 10^{-1}$	$1.31\times 10^{-1}$	$6.36\times 10^{-1}$	$9.93\times 10^{-1}$	$5.35\times 10^{-2}$	$8.58\times 10^{-1}$
**Cow11**	$9.90\times 10^{-1}$	$4.91\times 10^{-1}$	$3.54\times 10^{-1}$	$6.56\times 10^{-2}$	$4.76\times 10^{-1}$	$7.82\times 10^{-1}$	$9.17\times 10^{-1}$	$7.49\times 10^{-1}$	$5.43\times 10^{-1}$	$5.35\times 10^{-1}$	NA	$3.29\times 10^{-1}$	$3.98\times 10^{-1}$	$8.45\times 10^{-1}$	$5.84\times 10^{-1}$	$2.29\times 10^{-1}$	$5.90\times 10^{-1}$
**Cow12**	$4.12\times 10^{-1}$	$7.20\times 10^{-1}$	$7.34\times 10^{-2}$	$5.55\times 10^{-1}$	$9.61\times 10^{-2}$	$2.56\times 10^{-1}$	$4.23\times 10^{-1}$	$2.19\times 10^{-1}$	$5.21\times 10^{-1}$	$1.02\times 10^{-1}$	$3.29\times 10^{-1}$	NA	$8.14\times 10^{-1}$	$2.71\times 10^{-1}$	$1.67\times 10^{-1}$	$9.83\times 10^{-1}$	$2.15\times 10^{-1}$
**Cow13**	$4.88\times 10^{-1}$	$8.92\times 10^{-1}$	$6.77\times 10^{-2}$	$2.73\times 10^{-1}$	$8.39\times 10^{-2}$	$3.08\times 10^{-1}$	$5.04\times 10^{-1}$	$2.81\times 10^{-1}$	$7.77\times 10^{-1}$	$1.31\times 10^{-1}$	$3.98\times 10^{-1}$	$8.14\times 10^{-1}$	NA	$3.91\times 10^{-1}$	$1.69\times 10^{-1}$	$7.02\times 10^{-1}$	$1.17\times 10^{-1}$
**Cow14**	$8.69\times 10^{-1}$	$4.35\times 10^{-1}$	$5.18\times 10^{-1}$	$5.07\times 10^{-2}$	$5.84\times 10^{-1}$	$9.24\times 10^{-1}$	$7.78\times 10^{-1}$	$9.41\times 10^{-1}$	$5.32\times 10^{-1}$	$6.36\times 10^{-1}$	$8.45\times 10^{-1}$	$2.71\times 10^{-1}$	$3.91\times 10^{-1}$	NA	$7.42\times 10^{-1}$	$1.95\times 10^{-1}$	$6.67\times 10^{-1}$
**Cow15**	$6.21\times 10^{-1}$	$2.23\times 10^{-1}$	$7.39\times 10^{-1}$	$2.36\times 10^{-2}$	$9.30\times 10^{-1}$	$8.10\times 10^{-1}$	$5.17\times 10^{-1}$	$8.06\times 10^{-1}$	$2.19\times 10^{-1}$	$9.93\times 10^{-1}$	$5.84\times 10^{-1}$	$1.67\times 10^{-1}$	$1.69\times 10^{-1}$	$7.42\times 10^{-1}$	NA	$9.00\times 10^{-2}$	$9.97\times 10^{-1}$
**Cow16**	$2.98\times 10^{-1}$	$6.15\times 10^{-1}$	$3.07\times 10^{-2}$	$4.72\times 10^{-1}$	$4.03\times 10^{-2}$	$1.64\times 10^{-1}$	$2.99\times 10^{-1}$	$1.38\times 10^{-1}$	$4.25\times 10^{-1}$	$5.35\times 10^{-2}$	$2.29\times 10^{-1}$	$9.83\times 10^{-1}$	$7.02\times 10^{-1}$	$1.95\times 10^{-1}$	$9.00\times 10^{-2}$	NA	$8.67\times 10^{-2}$
**Cow17**	$6.39\times 10^{-1}$	$2.08\times 10^{-1}$	$7.07\times 10^{-1}$	$2.86\times 10^{-2}$	$9.77\times 10^{-1}$	$7.57\times 10^{-1}$	$5.21\times 10^{-1}$	$8.36\times 10^{-1}$	$1.33\times 10^{-1}$	$8.58\times 10^{-1}$	$5.90\times 10^{-1}$	$2.15\times 10^{-1}$	$1.17\times 10^{-1}$	$6.67\times 10^{-1}$	$9.97\times 10^{-1}$	$8.67\times 10^{-2}$	NA
**Randomized data 10**																	
	**Cow01**	**Cow02**	**Cow03**	**Cow04**	**Cow05**	**Cow06**	**Cow07**	**Cow08**	**Cow09**	**Cow10**	**Cow11**	**Cow12**	**Cow13**	**Cow14**	**Cow15**	**Cow16**	**Cow17**
**Cow01**	NA	$4.38\times 10^{-1}$	$3.61\times 10^{-1}$	$6.01\times 10^{-1}$	$8.94\times 10^{-1}$	$4.81\times 10^{-1}$	$4.55\times 10^{-1}$	$3.43\times 10^{-1}$	$3.14\times 10^{-2}$	$8.19\times 10^{-1}$	$2.49\times 10^{-1}$	$6.01\times 10^{-1}$	$7.87\times 10^{-1}$	$5.72\times 10^{-1}$	$2.44\times 10^{-1}$	$5.35\times 10^{-1}$	$1.15\times 10^{-1}$
**Cow02**	$4.38\times 10^{-1}$	NA	$8.33\times 10^{-2}$	$2.00\times 10^{-1}$	$4.17\times 10^{-1}$	$1.00\times 10^{0}$	$1.35\times 10^{-1}$	$9.63\times 10^{-2}$	$2.71\times 10^{-3}$	$6.28\times 10^{-1}$	$3.50\times 10^{-2}$	$8.62\times 10^{-1}$	$1.99\times 10^{-1}$	$7.54\times 10^{-1}$	$5.98\times 10^{-2}$	$1.09\times 10^{-1}$	$1.01\times 10^{-2}$
**Cow03**	$3.61\times 10^{-1}$	$8.33\times 10^{-2}$	NA	$7.11\times 10^{-1}$	$3.85\times 10^{-1}$	$8.05\times 10^{-2}$	$8.53\times 10^{-1}$	$9.80\times 10^{-1}$	$1.88\times 10^{-1}$	$2.18\times 10^{-1}$	$7.98\times 10^{-1}$	$1.24\times 10^{-1}$	$4.99\times 10^{-1}$	$1.25\times 10^{-1}$	$7.59\times 10^{-1}$	$7.47\times 10^{-1}$	$5.39\times 10^{-1}$
**Cow04**	$6.01\times 10^{-1}$	$2.00\times 10^{-1}$	$7.11\times 10^{-1}$	NA	$6.58\times 10^{-1}$	$2.14\times 10^{-1}$	$8.53\times 10^{-1}$	$6.95\times 10^{-1}$	$1.09\times 10^{-1}$	$4.42\times 10^{-1}$	$5.17\times 10^{-1}$	$2.73\times 10^{-1}$	$8.41\times 10^{-1}$	$2.86\times 10^{-1}$	$5.49\times 10^{-1}$	$8.72\times 10^{-1}$	$2.85\times 10^{-1}$
**Cow05**	$8.94\times 10^{-1}$	$4.17\times 10^{-1}$	$3.85\times 10^{-1}$	$6.58\times 10^{-1}$	NA	$3.86\times 10^{-1}$	$5.16\times 10^{-1}$	$4.03\times 10^{-1}$	$3.67\times 10^{-2}$	$7.18\times 10^{-1}$	$2.55\times 10^{-1}$	$5.22\times 10^{-1}$	$8.03\times 10^{-1}$	$5.52\times 10^{-1}$	$2.87\times 10^{-1}$	$5.26\times 10^{-1}$	$9.64\times 10^{-2}$
**Cow06**	$4.81\times 10^{-1}$	$1.00\times 10^{0}$	$8.05\times 10^{-2}$	$2.14\times 10^{-1}$	$3.86\times 10^{-1}$	NA	$1.30\times 10^{-1}$	$8.58\times 10^{-2}$	$3.35\times 10^{-3}$	$6.33\times 10^{-1}$	$4.00\times 10^{-2}$	$8.22\times 10^{-1}$	$2.15\times 10^{-1}$	$7.91\times 10^{-1}$	$6.16\times 10^{-2}$	$1.23\times 10^{-1}$	$8.74\times 10^{-3}$
**Cow07**	$4.55\times 10^{-1}$	$1.35\times 10^{-1}$	$8.53\times 10^{-1}$	$8.53\times 10^{-1}$	$5.16\times 10^{-1}$	$1.30\times 10^{-1}$	NA	$8.61\times 10^{-1}$	$1.45\times 10^{-1}$	$3.17\times 10^{-1}$	$6.79\times 10^{-1}$	$1.92\times 10^{-1}$	$6.30\times 10^{-1}$	$1.89\times 10^{-1}$	$6.42\times 10^{-1}$	$9.26\times 10^{-1}$	$4.00\times 10^{-1}$
**Cow08**	$3.43\times 10^{-1}$	$9.63\times 10^{-2}$	$9.80\times 10^{-1}$	$6.95\times 10^{-1}$	$4.03\times 10^{-1}$	$8.58\times 10^{-2}$	$8.61\times 10^{-1}$	NA	$2.11\times 10^{-1}$	$2.22\times 10^{-1}$	$7.97\times 10^{-1}$	$1.39\times 10^{-1}$	$5.44\times 10^{-1}$	$1.37\times 10^{-1}$	$7.90\times 10^{-1}$	$7.88\times 10^{-1}$	$4.59\times 10^{-1}$
**Cow09**	$3.14\times 10^{-2}$	$2.71\times 10^{-3}$	$1.88\times 10^{-1}$	$1.09\times 10^{-1}$	$3.67\times 10^{-2}$	$3.35\times 10^{-3}$	$1.45\times 10^{-1}$	$2.11\times 10^{-1}$	NA	$1.43\times 10^{-2}$	$2.72\times 10^{-1}$	$5.26\times 10^{-3}$	$3.71\times 10^{-2}$	$4.38\times 10^{-3}$	$3.47\times 10^{-1}$	$9.17\times 10^{-2}$	$4.27\times 10^{-1}$
**Cow10**	$8.19\times 10^{-1}$	$6.28\times 10^{-1}$	$2.18\times 10^{-1}$	$4.42\times 10^{-1}$	$7.18\times 10^{-1}$	$6.33\times 10^{-1}$	$3.17\times 10^{-1}$	$2.22\times 10^{-1}$	$1.43\times 10^{-2}$	NA	$1.25\times 10^{-1}$	$7.77\times 10^{-1}$	$4.95\times 10^{-1}$	$8.07\times 10^{-1}$	$1.60\times 10^{-1}$	$3.13\times 10^{-1}$	$4.33\times 10^{-2}$
**Cow11**	$2.49\times 10^{-1}$	$3.50\times 10^{-2}$	$7.98\times 10^{-1}$	$5.17\times 10^{-1}$	$2.55\times 10^{-1}$	$4.00\times 10^{-2}$	$6.79\times 10^{-1}$	$7.97\times 10^{-1}$	$2.72\times 10^{-1}$	$1.25\times 10^{-1}$	NA	$6.31\times 10^{-2}$	$3.47\times 10^{-1}$	$5.92\times 10^{-2}$	$9.63\times 10^{-1}$	$5.38\times 10^{-1}$	$6.96\times 10^{-1}$
**Cow12**	$6.01\times 10^{-1}$	$8.62\times 10^{-1}$	$1.24\times 10^{-1}$	$2.73\times 10^{-1}$	$5.22\times 10^{-1}$	$8.22\times 10^{-1}$	$1.92\times 10^{-1}$	$1.39\times 10^{-1}$	$5.26\times 10^{-3}$	$7.77\times 10^{-1}$	$6.31\times 10^{-2}$	NA	$3.35\times 10^{-1}$	$9.30\times 10^{-1}$	$8.59\times 10^{-2}$	$1.81\times 10^{-1}$	$1.79\times 10^{-2}$
**Cow13**	$7.87\times 10^{-1}$	$1.99\times 10^{-1}$	$4.99\times 10^{-1}$	$8.41\times 10^{-1}$	$8.03\times 10^{-1}$	$2.15\times 10^{-1}$	$6.30\times 10^{-1}$	$5.44\times 10^{-1}$	$3.71\times 10^{-2}$	$4.95\times 10^{-1}$	$3.47\times 10^{-1}$	$3.35\times 10^{-1}$	NA	$3.16\times 10^{-1}$	$3.41\times 10^{-1}$	$7.80\times 10^{-1}$	$1.73\times 10^{-1}$
**Cow14**	$5.72\times 10^{-1}$	$7.54\times 10^{-1}$	$1.25\times 10^{-1}$	$2.86\times 10^{-1}$	$5.52\times 10^{-1}$	$7.91\times 10^{-1}$	$1.89\times 10^{-1}$	$1.37\times 10^{-1}$	$4.38\times 10^{-3}$	$8.07\times 10^{-1}$	$5.92\times 10^{-2}$	$9.30\times 10^{-1}$	$3.16\times 10^{-1}$	NA	$8.84\times 10^{-2}$	$1.81\times 10^{-1}$	$1.83\times 10^{-2}$
**Cow15**	$2.44\times 10^{-1}$	$5.98\times 10^{-2}$	$7.59\times 10^{-1}$	$5.49\times 10^{-1}$	$2.87\times 10^{-1}$	$6.16\times 10^{-2}$	$6.42\times 10^{-1}$	$7.90\times 10^{-1}$	$3.47\times 10^{-1}$	$1.60\times 10^{-1}$	$9.63\times 10^{-1}$	$8.59\times 10^{-2}$	$3.41\times 10^{-1}$	$8.84\times 10^{-2}$	NA	$5.75\times 10^{-1}$	$7.62\times 10^{-1}$
**Cow16**	$5.35\times 10^{-1}$	$1.09\times 10^{-1}$	$7.47\times 10^{-1}$	$8.72\times 10^{-1}$	$5.26\times 10^{-1}$	$1.23\times 10^{-1}$	$9.26\times 10^{-1}$	$7.88\times 10^{-1}$	$9.17\times 10^{-2}$	$3.13\times 10^{-1}$	$5.38\times 10^{-1}$	$1.81\times 10^{-1}$	$7.80\times 10^{-1}$	$1.81\times 10^{-1}$	$5.75\times 10^{-1}$	NA	$3.20\times 10^{-1}$
**Cow17**	$1.15\times 10^{-1}$	$1.01\times 10^{-2}$	$5.39\times 10^{-1}$	$2.85\times 10^{-1}$	$9.64\times 10^{-2}$	$8.74\times 10^{-3}$	$4.00\times 10^{-1}$	$4.59\times 10^{-1}$	$4.27\times 10^{-1}$	$4.33\times 10^{-2}$	$6.96\times 10^{-1}$	$1.79\times 10^{-2}$	$1.73\times 10^{-1}$	$1.83\times 10^{-2}$	$7.62\times 10^{-1}$	$3.20\times 10^{-1}$	NA
